# Phylogeny and Taxonomic Synopsis of the Genus *Bougainvillea* (Nyctaginaceae)

**DOI:** 10.3390/plants11131700

**Published:** 2022-06-27

**Authors:** Mary Ann C. Bautista, Yan Zheng, David E. Boufford, Zhangli Hu, Yunfei Deng, Tao Chen

**Affiliations:** 1 Key Laboratory of Plant Resource Conservation and Sustainable Utilization, South China Botanical Garden, Chinese Academy of Sciences, Guangzhou 510650, China; bautista.maryann@gmail.com; 2 Key Laboratory of South Subtropical Plant Diversity, Fairy Lake Botanical Garden, Chinese Academy of Sciences, Shenzhen 518004, China; 3 International School, University of Chinese Academy of Sciences, Beijing 100049, China; 4 Center for Conservation Innovations Ph, Inc., Alabang, Muntinlupa City 1770, Philippines; 5 Guangdong Institute of Traditional Chinese Medicine, Guangzhou 510520, China; zhengyan@gdtcm.org.cn; 6 Harvard University Herbaria, 22 Divinity Ave., Cambridge, MA 02138, USA; david_boufford@harvard.edu; 7 School of Life Sciences and Oceanology, Shenzhen University, Shenzhen 518055, China; huzl@szu.edu.cn

**Keywords:** *Bougainvillea*, Nyctaginaceae, chloroplast genome, phylogeny, morphology, nomenclature, taxonomy

## Abstract

*Bougainvillea* Comm. ex Juss. is one of the renowned genera in the Nyctaginaceae, but despite its recognized horticultural value, the taxonomy and phylogeny of the genus is not well-studied. Phylogenetic reconstructions based on plastid genomes showed that *B. pachyphylla* and *B. peruviana* are basal taxa, while *B. spinosa* is sister to two distinct clades: the predominantly cultivated *Bougainvillea* clade (*B. spectabilis*, *B. glabra*, *B. arborea*, *B.* cultivar, *B. praecox*) and the clade containing wild species of *Bougainvillea* (*B. berberidifolia*, *B. campanulata*, *B. infesta*, *B. modesta*, *B. luteoalba*, *B. stipitata*, and *B. stipitata* var. *grisebachiana*). Early divergence of *B. peruviana*, *B. pachyphylla* and *B. spinosa* is highly supported, thus the previously proposed division of *Bougainvillea* into two subgenera (*Bougainvillea* and *Tricycla*) was not reflected in this study. Morphological analysis also revealed that leaf arrangement, size, and indumentum together with the perianth tube and anthocarp shape and indumentum are important characteristics in differentiating the species of *Bougainvillea*. In the present study, 11 species and one variety are recognized in *Bougainvillea*. Six names are newly reduced to synonymy, and lectotypes are designated for 27 names. In addition, a revised identification key and illustrations of the distinguishing parts are also provided in the paper.

## 1. Introduction

*Bougainvillea* Comm. ex Juss. (Nyctaginaceae; Bouginvilleeae) is a commonly cultivated plant group with colorful bracts in the four o’clock family. The type species, *Bougainvillea spectabilis*, was discovered by the French botanist Philibert Commerson (with his assistant Jean Baret) in Rio de Janeiro, Brazil, during the 1760s [1]. Commerson was the botanist accompanying French Navy Admiral Louis-Antoine de Bougainville on the voyage to circumnavigate the Earth. Since de Bougainville was the commander of the voyage, the newly discovered genus was named after him. The genus name, originally spelled *Bugainvillaea* Jussieu [2], has many orthographic variants. Spach [3] was the first to adopt the spelling *Bougainvillea*, which was later conserved [4,5] and listed in Appendix III of the *International Code of Nomenclature for algae*, *fungi*, *and plants* (Shenzhen Code) [6]. Most species of *Bougainvillea* were published by Heimerl [7], Standley [8,9,10], and Toursarkissian [11]. Based on previous publications, 14–18 species of *Bougainvillea* have been recognized, although the basis for separating the species and the differences between them may be inconsequential since they are highly similar in appearance [8]. We recognize 11 species here.

Morphologically, plants of *Bougainvillea* are scandent shrubs or small trees often armed with simple or forked thorns. The colorful structures often mistaken as flowers are actually modified bracts surrounding small tubular flowers. The flower is usually attached to the inner surface of each bract and its pedicel is confluent with the midrib of the bract [12]. Based mainly on the branching of the thorn apex, Standley [10] divided *Bougainvillea* into two subgenera: subg. *Tricycla* and subg. *Eubougainvillea*. According to Art. 21.3 and 22.2 of the Shenzhen Code [6], *Eubougainvillea* may be corrected to *Bougainvillea*. Only *Bougainvillea spinosa* with furcate or forked thorns was categorized into subg. *Tricycla*, while the remaining species were classified into subg. *Bougainvillea*. Bract size and color were considered important characters in subdividing *Bougainvillea* into two unnamed groups. The first group, with large (ca. 2.5 to 4 cm long), brightly colored bracts, contains *B. pachyphylla*, *B. peruviana*, *B. glabra*, and *B. spectabilis*. The second group, which has smaller (<2.5 cm long) and less conspicuous bracts or sometimes brightly colored bracts that do not retain their color as they dry, contains *B. berberidifolia*, *B. campanulata*, *B. infesta*, *B. praecox*, and *B. stipitata* [8,10]. This proposed subdivision was based entirely on morphological characteristics. 

In previous phylogenetic studies of the Caryophyllales, *Bougainvillea* together with other genera in Nyctaginaceae was placed in the phytolaccoid clade of a larger ‘globular inclusion’ clade [13,14,15]. The molecular phylogeny of Nyctaginaceae [16] based on three plastid genes (*ndh*F, *rps*16, *rpl*16) and one nuclear region (nrITS) significantly developed the understanding of the relationships within the family, ensuing the reevaluation of the tribal classification [17]. Although Douglas and Manos [16] involved nearly all genera of Nyctaginaceae in their study, only partial sequences of *Bougainvillea glabra* and *B. infesta* were included. Douglas and Manos [16] fully resolved the position of *Bougainvillea* within Nyctaginaceae as sister to *Belemia* and *Phaeoptilum*, but the relationship among the species of *Bougainvillea* was not established in their paper. A recent study on the plastid genomes of some wild and cultivated plants of *Bougainvillea* showed that *B. peruviana* and *B. pachyphylla* diverged earlier than other species of *Bougainvillea*, while the commonly known *B. glabra* clustered with *B. spectabilis* and a *B.* cultivar [18]. Since only a few samples were included in the analysis, limited information about the relationship among the species of *Bougainvillea* was inferred. Thus, the current study reported here sought to describe the phylogenetic relationships within *Bougainvillea* and to provide a taxonomic synopsis of the genus.

## 2. Results

### 2.1. Sequence Characteristics

**Figure 1:**
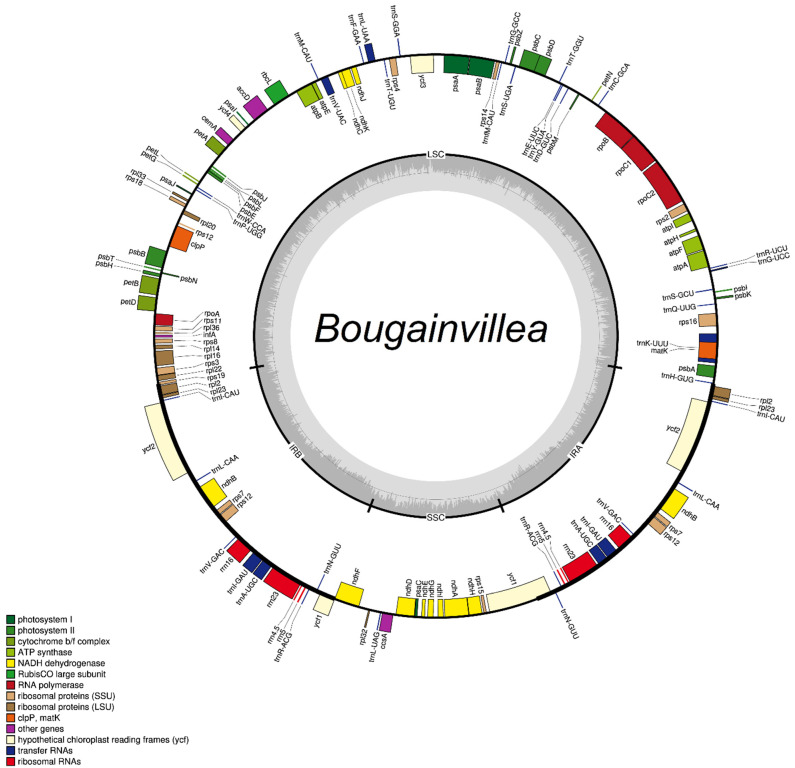
General circular gene map of *Bougainvillea* chloroplast genomes showing the large single-copy (LSC) region, small single-copy (SSC) region, and inverted repeat regions (IRA, IRB). Genes within the circle were also color-coded according to their functional group. The dark gray and light gray plots correspond to the GC and AT contents, respectively.

The plastid genomes of *Bougainvillea berberidifolia*, *B. campanulata*, *B. infesta*, *B. arborea*, *B. modesta*, *B. luteoalba*, *B. spinosa*, *B. stipitata*, *B. stipitata* var. *grisebachiana* together with the previously sequenced *B. glabra*, *B. peruviana*, *B. pachyphylla*, *B. praecox*, *B. spectabilis*, and *B*. cultivar had sequence lengths ranging from 153,966 bp to 154,872 bp. As in most flowering plants, they all possessed the usual quadripartite structure composed of 85,159–85,958 bp large single-copy (LSC) region, 17,997–18,078 bp small single-copy (SSC) region, and 25,377–25,503 bp pair of inverted repeats (Figure 1, Table S1). Although there were differences in size, and *B. spinosa* had the largest genome, all sequenced plastid genomes of *Bougainvillea* had 131 genes, consisting of 86 protein-coding genes, 37 transfer RNA (tRNA) genes, and eight ribosomal RNA (rRNA) genes (Table S2). Of these genes, 18 genes were duplicated (*ndh*B, *rp*l2, *rp*l23, *rps*7, *rps*12, *ycf*1, *ycf*2, *rrn*4.5, *rrn*5, *rrn*16, *rrn*23, *trn*I-CAU, *trn*L-CAA, *trn*V-GAC, *trn*I-GAU, *trn*A-UGC, *trn*R-ACG, *trn*N-GUU) in the inverted repeat regions. There were also 17 intron-containing genes, of which 15 genes (*rps*16, *atp*F, *rpo*C1, *pet*B, *pet*D, *rp*l16, *rp*l2, *ndh*B, *ndh*A, *trn*I-GAU, *trn*A-UGC, *trn*V-UAC, *trn*L-UAA, *trn*G-UCC, *trn*K-UUU) had a single intron; clpP and ycf3 had two introns each. The GC content of each chloroplast genome was highly similar as well, ranging from 36.4% to 36.6%.

### 2.2. SNPs and Indels Analysis

**Figure 2:**
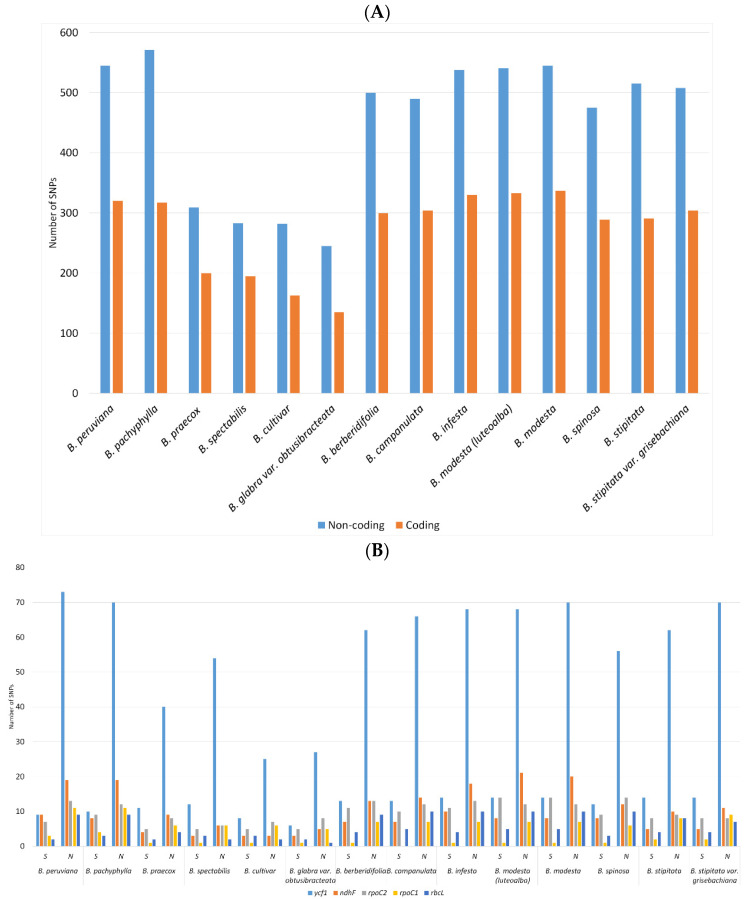
Total number of SNPs identified in the plastid genomes of *Bougainvillea*. (**A**) Number SNPs observed in the coding and non-coding sequences. (**B**) Coding sequences with greatest occurrence of synonymous and non-synonymous SNPs.

The SNPs (single nucleotide polymorphisms) and indels (insertions-deletions) were identified in the plastid genomes of *Bougainvillea* using MUMmer4 [19] and Geneious Prime 2020.1 [20]. The overall results indicated that there were more SNPs and indels in the chloroplast genomes of *B. pachyphylla*, *B. peruviana*, and *B. modesta*. This can be attributed to the fact that the three aforementioned species were distantly related to the reference species, *B. glabra*. The non-coding sequences of *B. pachphylla* had 571 SNPs while the coding sequences had 317 SNPs. Correspondingly, there were 545 SNPs in the non-coding sequences and 337 SNPs in the coding sequences of *B. modesta* (Figure 2A). In contrast, *B. praecox* (309, 200), *B. spectabilis* (283, 195), *B. cultivar* (282, 163), and *B. arborea* (245, 135) had fewer SNPs, suggesting that those genomes are highly similar to the reference genome of *B. glabra*. In congruence with the preceding study [18], several protein-coding genes had a higher frequency of SNPs. The *ycf*1 reading frame had the greatest number of synonymous and non-synonymous SNPs in all samples of *Bougainvillea*. (Figure 2B). In addition, the genes for RNA polymerase (*rpo*A, *rpo*B, *rpo*C2, *rpo*C1), NADH-dehydrogenase (ndhF, ndhA), rubisco (rbcL), maturase (matK), and hypothetical reading frames (*ycf*1, *ycf*2) contained relatively more synonymous and non-synonymous SNPs. The patterns of synonymous (Ks) and non-synonymous (Ka) substitutions also revealed that those genes are possibly under significant positive selection since they have Ka/Ks values > 1.

**Figure 3:**
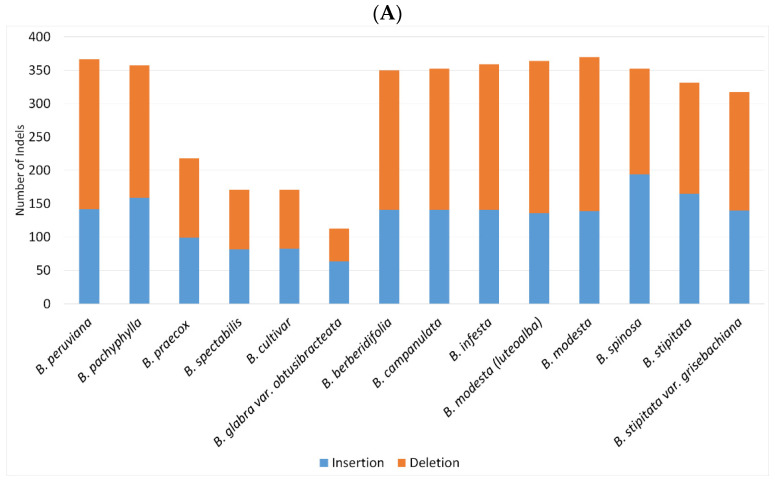

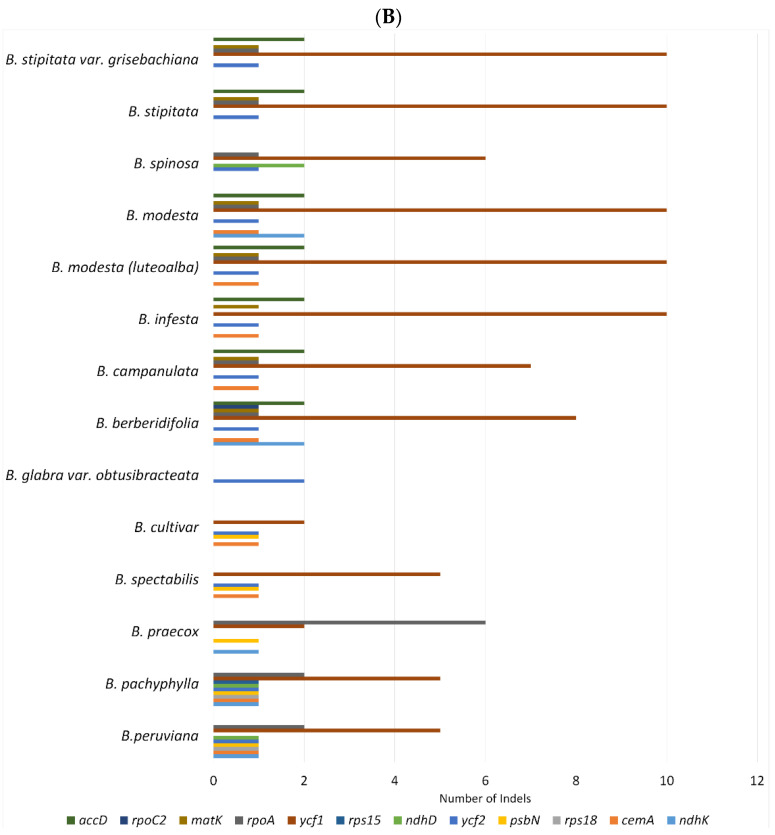
Total number of indels found in plastid genomes of *Bougainvillea*. (**A**) Number of indels observed in coding and non-coding sequences. (**B**) Coding genes with greatest occurrence of insertions and deletions.

In comparison, *B. peruviana* (366) and *B. modesta* (370) had the most insertions and deletions, while *B. praecox* (218), *B. spectabilis* (171), *B. cultivar* (171), and *B. arborea* (113) had fewer indels (Figure 3A). *Bougainvillea arborea* had the fewest indels, denoting fewer differences from the reference species, *B. glabra*. The presence of large indels (16-55 bp) in the *clp*P introns of almost all species mainly differentiated *B. glabra* from other species. Both *B. peruviana* and *B. pachyphylla* differed as well from *B. glabra* by the large deletion (43 bp) between the *rpl*22 and *rps*19 genes (Figure S1). When compared to *B. glabra*, several deletions were observed in most wild species of *Bougainvillea* (Figure S1). A 16-bp deletion was discovered in the *rps*16 introns of *B. berberidifolia*, *B. campanulata*, *B. infesta*, *B. modesta*, *B. luteoalba*, *B. stipitata* and *B. stipitata* var. *grisebachiana*. Additionally, deletions in the *trn*R-AGC–*trn*N-GUU (28 bp) and *rp*l32–*trn*L-UAG (36–42 bp) spacers were detected in the wild species of *Bougainvillea* (Figure S1). Even though small indels are more common in the non-coding sequences, small deletions were found in the *mat*K (6 bp) and *acc*D (9 bp and 6 bp) genes of the wild species of *Bougainvillea* (Figure S1). A large number of indels were also found in the *ycf*1 genes of all cp genomes of *Bougainvillea* (Figure 3B).

### 2.3. Phylogenetic Analysis

**Figure 4:**
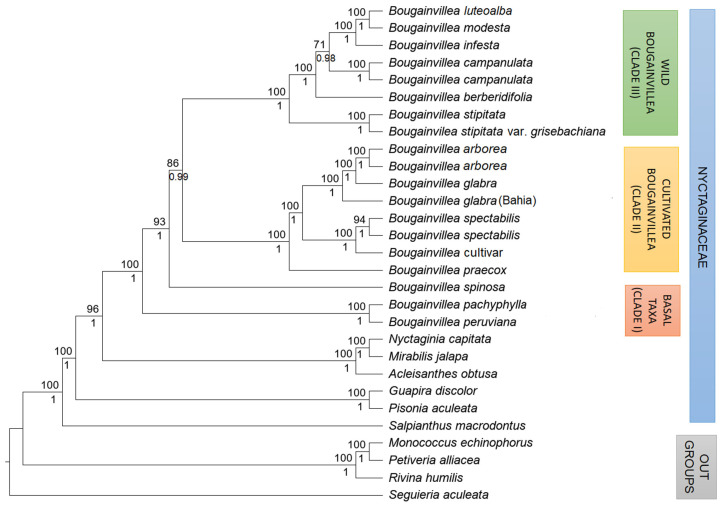
Maximum Likelihood (ML) and Bayesian Inference (BI) phylogenetic tree based on alignment of 79 protein-coding genes of 25 Nyctaginaceae chloroplast genomes. Numbers above nodes represent ML bootstrap values while numbers below nodes are Bayesian posterior probability values.

Phylogenetic trees reconstructed with Bayesian Inference (BI) and Maximum Likelihood (ML) analyses resulted in congruent topologies and differed only in support values. In general, *Bougainvillea* (Bougainvilleeae) showed a closer relationship to *Acleisanthes*, *Mirabilis*, and *Nyctaginia* (Nyctagineae) than to any other genera in Nyctaginaceae included in this study (Figure 4). Within *Bougainvillea*, *B. peruviana* and *B. pachyphylla* were the basal-most taxa (clade I) while *B. spinosa* was sister to two well-defined clades: clade II or the predominantly ‘cultivated’ *Bougainvillea* clade (*B. spectabilis*, *B. glabra*, *B. arborea*, *B.* cultivar, *B. praecox*) and clade III or the ‘wild’ *Bougainvillea* clade (*B. berberidifolia*, *B. campanulata*, *B. infesta*, *B. modesta*, *B. luteoalba*, *B. stipitata*, *B. stipitata* var. *grisebachiana*).

In the predominantly cultivated *Bougainvillea* group (clade II), the inconspicuous *Bougainvillea praecox* was sister to two apparent subclades containing *B. glabra* and *B. spectabilis* (BS = 100, BPP = 1) (Figure 4). Within the ‘*glabra*’ subclade, *B. arborea* samples were grouped together with *B. glabra* (BS = 100, BPP = 1). In contrast, the *Bougainvillea* cultivar representative was clustered with *B. spectabilis* (BS = 100, BPP = 1). Clade III, composed mainly of wild species, displayed no specific grouping pattern. *Bougainvillea stipitata* was in the basal position, a grade higher than the remaining wild species of *Bougainvillea*. *Bougainvillea berberidifolia*, *B. campanulata*, *B. infesta*, and *B. modesta* subsequently followed *B. stipitata*. *Bougainvillea modesta* had a closer relationship to *B. infesta* than with the rest of *Bougainvillea* (BS = 100, BPP = 1).

## 3. Discussion

**Figure 5:**
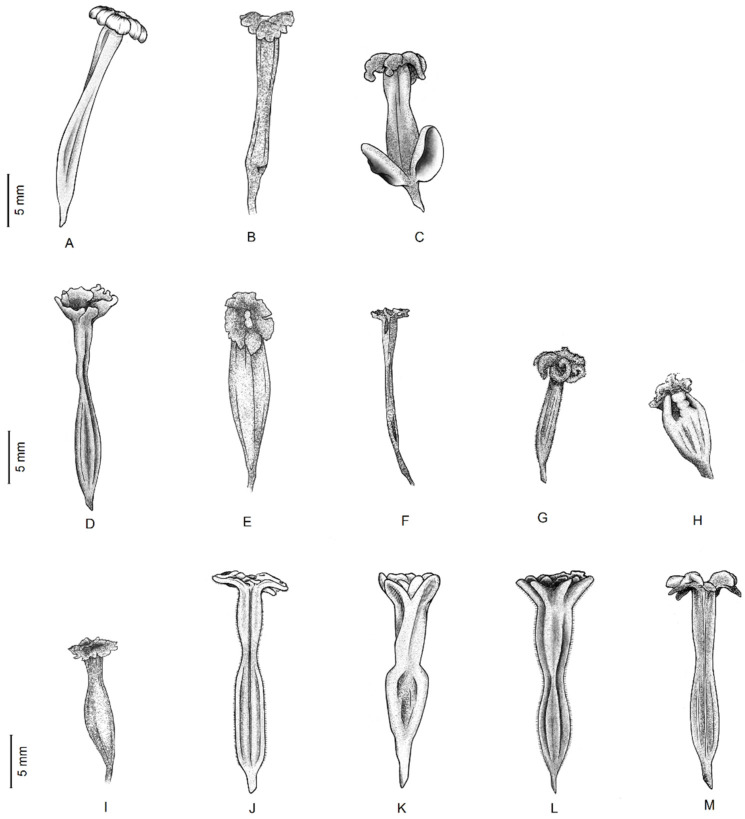
Perianth tubes of species and cultivars of *Bougainvillea*: basal taxa: (**A**) *Bougainvillea peruviana*, (**B**) *B. pachyphylla*; (**C**) *B. spinosa*; wild *Bougainvillea*: (**D**) *B. stipitata*, (**E**) *B. infesta*, (**F**) *B. berberidifolia*, (**G**) *B. modesta*, (**H**) *B. campanulata*; cultivated *Bougainvillea***:** (**I**) *B. praecox*, (**J**) *B. glabra*, (**K**) *B. arborea*, (**L**) *B. spectabilis*, and (**M**) *B.* cultivar. (Illustrated by Eingel Cannerie Jaramillo).

The plastid-based phylogeny obtained in this analysis strongly supported the early divergence of both *Bougainvillea peruviana* and *B. pachyphylla*. Consistent with a prior study [18], these two are considered the basal-most species of *Bougainvillea*. In morphological features, *B. peruviana* was associated with either *B. glabra* [8,21] or *B. pachyphylla* [10], but genetic information from plastid genomes confirmed a higher affinity with *B. pachyphylla* [18]. The analyses of SNPs and indels presented in this study also signified that *B. peruviana* and *B. pachyphylla* represented two genomes distinct from *B. glabra*. Both species are morphologically similar except in leaf texture and perianth indumentum. *Bougainvillea peruviana* has a thin leaf blade and a glabrous perianth in contrast to the thick leathery leaf blade and densely puberulent perianth of *B. pachyphylla* [9,10]. Both *B. peruviana* and *B. pachyphylla* have a slender, almost linear-oblong perianth tube but the tube of the latter is a bit wider near the perianth lobes (Figure 5). Early divergence of these two taxa also suggests that the two subgenera (*Tricycla* and *Bougainvillea*) classification of Standley [10] does not coincide with the current analysis. *Bougainvillea pachyphylla* and *B. peruviana* are not closely related to other members of subg. *Bougainvillea*. Though *B. pachyphylla* and *B. peruviana* have simple thorns similar to the other species of *Bougainvillea*, they are more basal than *B. spinosa* (subg. *Tricycla*), suggesting that the *Bougainvillea* cannot be subdivided based on thorn branching alone.

*Bougainvillea spinosa* differs from other species of *Bougainvillea* by having forked or furcate thorns [8]. Moreover, the solitary flower surrounded by three bracts and the thick, fleshy leaves arranged into brachyblasts makes it more morphologically distinct from other species. Consequently, earlier classifications treated *B. spinosa* as a single species of subgenus *Tricycla*. The molecular analysis did not concur with this classification, but it clearly showed that *B. spinosa* does not have a close relationship with other species of *Bougainvillea*. It is also not the basal-most taxon but diverged earlier than the two major clades of *Bougainvillea*, the ‘cultivated’ *Bougainvillea* group (clade II) and the ‘wild’ *Bougainvillea* group (clade III). 

Initially, it was assumed that *B. praecox* was synonymous with *B. modesta* due to similarities in appearance and lack of distinguishing characteristics, but plastid genome data showed that it has a closer relationship with the ornamental species, such as *B. glabra* and *B. spectabilis*. Sequence variation analysis further supported the close relationship of *B. praecox* to the cultivated *Bougainvillea*. High sequence similarity was observed between *B. praecox* and the reference *B. glabra*. In contrast, *B. modesta* had the greatest variation in sequences when compared to *B. glabra*, implying that *B. modesta* is not a close relative of *B. glabra*.

**Figure 6:**
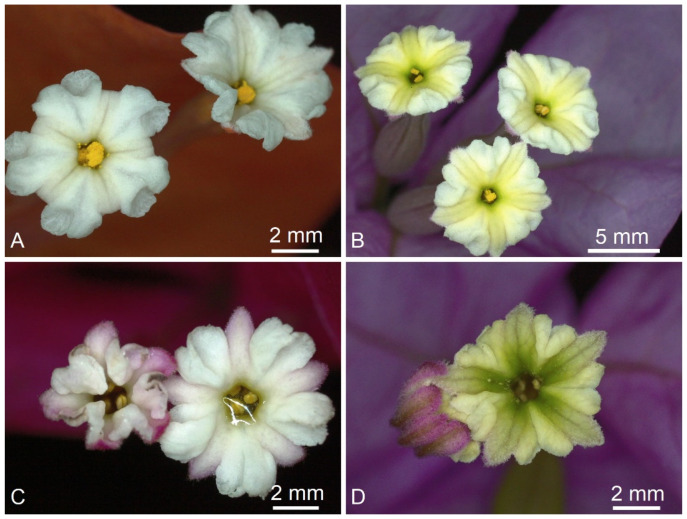
Perianth lobes of species of *Bougainvillea* and a cultivar: (**A**) *Bougainvillea peruviana*, (**B**) *B. glabra*, (**C**) *B.* cultivar, and (**D**) *B. arborea*.

The sister-group relationship between the *Bougainvillea glabra* subclade and the *B. spectabilis* subclade was already established, since *B. glabra* and the cultivars are hardly differentiated from *B. spectabilis* [8]. Both the ‘*glabra*’ and ‘*spectabilis*’ subclades have thin, alternate leaves and large (2.5 to 4.5 cm), colorful, acute or acuminate bracts, and a constricted perianth tube (Figure 5). Members of the ‘*glabra*’ subclade typically have glabrate to puberulent vegetative parts while the ‘*spectabilis*’ subclade can be characterized by having a fulvous to villous stem and a villous abaxial leaf surface [8,10]. Sequences deposited in GenBank are mostly from cultivated plants and are identified as either *B. glabra* or *B. spectabilis*. In the *B. glabra* group, it was quite evident that the samples identified as *B. arborea* were closely associated to *B. glabra*. *Bougainvillea arborea* might be distinct from *B. glabra* by its tree-like habit (vs. scandent shrub), unarmed or sparsely armed with simple thorns (vs. armed with simple stout thorns), greenish-yellow perianth lobes (vs. yellowish-white or cream perianth lobes) (Figure 6), and obconical-obturbinoid or fusiform (vs. oblong) anthocarp and base of the perianth tube (Figure 5). Further studies are needed to validate the exact relationship between *B. arborea* and *B. glabra*. On the other hand, *Bougainvillea* cultivar was within the *‘spectabilis*’ group, since cultivars are usually crossed between the two species, *B. glabra* and *B. spectabilis*. Thereby, it is expected that most cultivars will be closer to either *B. glabra* or *B. spectabilis*.

**Figure 7:**
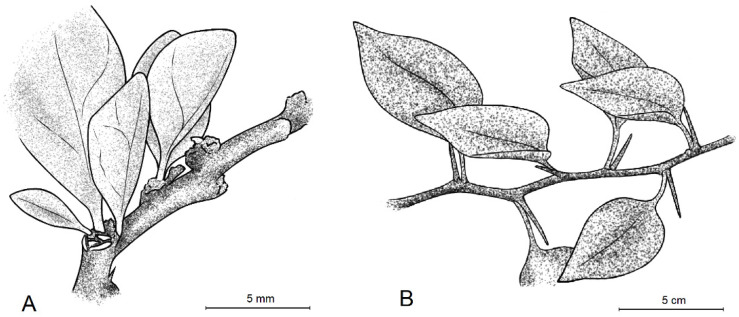
Leaf arrangement of *Bougainvillea berberidifolia* (**A**) and *Bougainvillea stipitata* (**B**). (Illustrated by Eingel Cannerie Jaramillo).

The majority of the wild species of *Bougainvillea* grouped together in clade III. The species of *Bougainvillea* (*B. berberidifolia*, *B. campanulata*, *B. infesta*) with thin leaves arranged into fascicles or brachyblasts are members of this group (Figure 7) [8,10]. Wild species of *Bougainvillea* with thin, alternate leaves such as *B. stipitata* and *B. modesta* also belong to clade III. Most of these wild species have smaller and unostentatious, normally white, greenish, or pale pink bracts, although the shade of color of the perianth lobes is more striking than in cultivated plants. Unlike the typical whitish or cream perianth lobes (Figure 6), wild species have brighter shades of green (*B. stipitata*, *B. infesta*), yellow (*B. campanulata*, *B. modesta*), or red (*B. berberidifolia*) [22,23].

Aside from the above-mentioned characteristics, there are no other unifying features that represent clade III. Perianth tubes are highly-variable and might be informative in differentiating wild species of *Bougainvillea*, but it is not a character that can be used to define the group (clade III). Thus, further morphological and anatomical studies may elucidate the relationships among the wild species of *Bougainvillea*. Nonetheless, analysis of SNPs and indels revealed high sequence similarities among the species. When aligned with *B. glabra*, large deletions were identified in the *rps*16 intron and a few intergenic spacers (*trn*R-AGC*–trn*N-GUU and *rpl*32*–trn*L-UA*G*) of all species in this clade. Small deletions were also noticeable in the *mat*K and *acc*D genes of these species. The deletions were not observed in sequences from the ‘cultivated’ *Bougainvillea* clade.

Based on our analyses, several taxonomic relationships could be established. The classification proposed by Standley [10] was not supported in this study based on chloroplast genomes. Early divergence of *Bougainvillea peruviana*, *B. pachyphylla*, and *B. spinosa* was highly supported, thus making them the basal taxa in *Bougainvillea*. Specifically, high morphological and molecular similarities were observed between *B. pachyphylla* and *B. peruviana* (clade I). The remaining species of *Bougainvillea* diverged into two monophyletic clades, the predominantly ‘cultivated’ *Bougainvillea* group (clade II) and the ‘wild’ *Bougainvillea* group (clade III). In addition, the present analyses did not support the previously proposed merging of *B. praecox* and *B. modesta*; *B. modesta* is clearly a species distinct from *B. praecox* as evidenced by the plastid genome data and perianth structure (Figure 5). *Bougainvillea praecox* is sister to *B. spectabilis* and *B. glabra*, while *B. modesta* belongs to the ‘wild’ *Bougainvillea* clade. The analyses also confirmed that *B. luteoalba* is a synonym of *B. modesta* and *B. stipitata* var. *grisebachiana* is a synonym of *B. stipitata*. The *B*. *arborea* samples fell into the clade of *B. glabra* and resulted in a new synonymy under *B. glabra* var. *obtusibracteata*. The results of this study have taxonomic implications in the classification of *Bougainvillea*. Enumerated here, therefore, are the names of the species of *Bougainvillea* (and their synonyms) that we accept.

### 3.1. Taxonomic Treatment

***Bougainvillea*** Comm. ex Juss., Gen. 91. 1789, nom. et orth. cons. ‘*Bugainvillaea*’. Type: *B. spectabilis* Willd., Sp. Pl. 2: 348. 1799, typ. cons.

*Tricycla* Cav., Icon. 6: 78. 1801. Type: *Tricycla spinosa* Cav.

*Josepha* Vell., Fl. Flumin.154. 1829. Type: *Josepha augusta* Vell.

Shrubs or small trees, often scandent, usually armed with simple or furcate thorns; leaves alternate or in brachyblasts, petiolate, entire; flowers perfect, either solitary and subtended by 3 bracts or typically in a 3-flowered, axillary inflorescence comprising 3 large, persistent, often brightly colored bracts with a flower borne on the inner surface of each bract, its pedicel confluent with the costa of the bract; perianth tubular, terete, usually constricted in the middle and ends in several induplicate-valvate or contorted lobes, the limb usually composed of glandular or non-glandular central lobes and adjacent commissural lobes; stamens 5–10, unequal to some extent, connate at the base into a cup-like structure; anthocarp woody, coriaceous, ribbed.

Distribution: Native to Argentina, Bolivia, Brazil, Ecuador, Paraguay, and Peru. Ornamental species (*Bougainvillea glabra* and *B. spectabilis*) introduced to most tropical and subtropical regions for cultivation.

1. Leaves less than 3 mm wide; inflorescences 1-flowered, surrounded by 3 bracts; thorns furcate or forked at apex..............***B. spinosa***1. Leaves more than 3 mm wide; inflorescences 3-flowered, surrounded by 3 bracts; thorns simple. 2. Bracts 2.5–4.5 cm long, usually strikingly colored; perianth lobes inconspicuous, white or cream.  3. Leaves thick, leathery .........................................................................................................................................................***B. pachyphylla***  3. Leaves thin and papery, especially when dry.   4. Leaves and perianth tube glabrous; apex of bracts usually obtuse or rounded...........................................................***B. peruviana***   4. Leaves and perianth tube variously pubescent; apex of bracts acute or acuminate.    5. Leaves densely villous; perianth tube short villous...................................................................................................***B. spectabilis***    5. Leaves merely puberulent; perianth tube sparsely to densely puberulent     6. Anthocarp oblong...............................................................................................................................................................***B. glabra***     6. Anthocarp obconical-obturbinoid to fusiform............................................................................***B. glabra* var**. ***obtusibracteata*** 2. Bracts 1–2.5 cm long, normally unostentatious; perianth lobes somewhat conspicuous, bright yellow, green, or red.  7. Perianth tube 6–7 mm long, campanulate, gradually widening from base to apex...................................................***B. campanulata***  7. Perianth tube 9–25 mm long, hypocrateriform or infundibuliform, slightly or highly constricted distally or in middle   8. Perianth tube ca. 1.5 mm wide, glabrous, reddish....................................................................................................***B. berberidifolia***   8. Perianth tube broader >1.5 mm wide, variously pubescent, greenish-brown or greenish-yellow.    9. Perianth tube 9–11 mm long, densely tomentulose     10. Perianth tube base ellipsoid, constricted in the middle...........................................................................................***B. praecox***     10. Perianth tube almost straight or slightly constricted..............................................................................................***B. modesta***    9. Perianth tube 12–25 mm long, densely pubescent or puberulent     11. Perianth tube densely pubescent; ovary navicular....................................................................................................***B. infesta***     11. Perianth tube puberulent; ovary spindle-shaped...................................................................................................***B. stipitata***

**1. *Bougainvillea peruviana*** Humb. & Bonpl., Pl. Aequinoct. 1: 174 t. 49. 1808, *“Bugainvillaea”*. ≡ Tricycla peruviana (Humb. & Bonpl.) Poir., Encycl. Suppl. 5: 359. 1817. Type: Peru, Colazai, *A.J.A. Bonpland & F.W.H.A. von Humboldt 3579* (lectotype designated here: P [P00670042]; isolectotypes: B [B10 0715477], MPU [MPU018935], P [P00712539, P00712540]). 

*Bougainvillea lehmanniana* Heimerl, Notizbl. Bot. Gart. Berlin-Dahlem 11: 466. 1932. Type: Ecuador, *F. C. Lehmann 255* (holotype, B [destroyed]), **syn. nov.**

Distribution: Peru, Ecuador.

Habitat: riverbanks, streams.

Note: The type (*F. C. Lehmann 255*) was the only recorded collection of *B. lehmanniana* from Ecuador. As no other specimen was collected and identified under this name, a neotype is not designated here.

Specimens examined: PERU. **Amazonas**: Utcubamba, *R. Vasquez et al. 25879* (F, MO); no precise locality, *H. van der Werff et al. 14639* (MO, US); **Cajamarca**: San Ignacio, *J. Campos de la Cruz & O. Díaz 2209* (MO, US); Celendin, *I. M. Sánchez Vega & J. G. Sánchez Vega 1936* (F); Jaén, *P. C. Hutchison 1415* (F, GH, MO, US); **La Libertad**: Bolivar, *R.W. Bussmannet al. 16697* (MO); **Tumbez**: Hacienda La Choza, *A. Weberbauer 7725* (F, US). ECUADOR. Loja to Macara, *T. Chen et al. 2014052606* (SZG).

**2**. ***Bougainvillea pachyphylla*** Heimerl ex Standl., Publ. Field Mus. Nat. Hist., Bot. Ser. 8(5): 308. 1931. Type: Peru, Piura, Oct. 1868, *A. Raimondi 8703* (holotype, B [destroyed], F photo negatives F0BN003094]); Peru, Piura, Prov. Ayabaca, Paimas, 10 Sept. 1976. *A. Sagástegui A. & J. Cabanillas S. 8726* (neotype designated here: U [U.1456198]; isoneotypes, MO [MO-2072063], HUT [HUT-13958]); Peru, Cajamarca, Chota, *A. Sagástegui A.* et al. *15924* (epitype, F [F2182839]).

Distribution: Ecuador, Peru

Habitat: near water courses.

Specimens examined: PERU. **Cajamarca**: Chota, *A. Sagástegui A. et al. 15924* (MO); **Paita**: Talara, *O. Haught 24* (F, US); **Piura**: Morropon, *A. Sagástegui A.*, *J. Cabanillas S. & O. Dios C. 8290* (MO, US); Ayabaca, A. *Sagástegui A. & J. Cabanillas S. 8726* (HUT, MO, U). ECUADOR. Loja, Celica, *H. Vargas*, *C. Canaday & R. Miranda 1174* (MO, QCNE, US). 

**3. *Bougainvillea spinosa*** (Cav.) Heimerl., Nat. Pflanzenfam. 3(1b): 27. 1889. ≡ *Tricycla spinosa* Cav., Icon. 6: 79, t. 598. 1798. Type: Argentina, *L. Née s.n.* (lectotype designated here: MA [MA652284])

*Bougainvillea patagonica* Decne., Voy. Amér. Mér. 8 (Bot. 1): t. 8. 1839. Type: [icon.] *Voy. Amér. Mér.* 8: Bot. I, pl. 8. 1848 (lectotype designated here).

*Tricycla spinosa* var. *parviflora* Phil., Anales Univ. Chile, Table 43: 534. 1873. ≡ *Bougainvillea spinosa* var. *parviflora* (Phil.) Heimerl, Conserv. Jard. Bot. Genéve 17: 231. 1913. Type: not located. Heimerl (Conserv. Jard. Bot. Genéve 17: 231. 1913) also did not see a specimen and knew it only from the description. He comments on var. *parviflora* “Die Pflanze wurde in den Provinz Mendoza [Argentina] bei Capis am Flusse Tunuyán, Dep. 9 Julio, von F. Leyboldt gesammelt; ich kenn sie aber nur aus der Beschriebung”.

*Bougainvillea patagonica* f. *eubracteata* Heimerl, Denkschr. Kaiserl. Akad. Wiss., Wien. Math.-Naturwiss. Kl. 70: 122. 1901. ≡ *Bougainvillea spinosa f. eubracteata* (Heimerl) Heimerl, Annuaire Conserv. Jard. Bot. Genéve 17: 230. 1913. Type: Not indicated in protologue and no specimen was cited.

*Bougainvillea patagonica* f. *microbracteata* Heimerl, Denkschr. Kaiserl. Akad. Wiss., Wien. Math.-Naturwiss. Kl. 70: 122. 1901. ≡ *Bougainvillea spinosa f. microbracteata* (Heimerl) Heimerl, Annuaire Conserv. Jard. Bot. Genéve 17: 230. 1913. Type: ARGENTINA, Mendoza, Cordillera do Paramillo, 1852, *J. Miers 565* (lectotype designated here: G [G00413530]).

*Bougainvillea spinosa* var. *conferta* Chodat et Wilczek, Bull. Herb. Boissier ser. 2, 2: 538. 1902. Type: ARGENTINA, Pampa de Sainl-Raphaël, buissons de 1 m. 50. 1897, *E. Wilczek 304* (lectotype designated here: LAU [LAU-0122620]; isolectotype: US [US03644381]).

Distribution: Argentina, Bolivia, upper Paraguay, and Peru.

Habitat: common on dry and rocky slopes.

Specimens examined: ARGENTINA. **Buenos Aires**, *L. Née s. n.* (MA); **La Pampa**: Caleu-caleu, *H. H. Bartlett 19943* (GH, US); **Mendoza**: Pampa de S. Rafael, *E. Wilczek 304* (US). **Río Negro**, *H. Senn 4325* (MO, US); Río Negro, General Roca, *W. Fischer 18* (GH, MO, US]). PERU. **Moquegua**: Torata, *A. Weberbauer 7414* (A, GH, US). BOLIVIA. **Chuquisaca**, ex herb. *Cardenasianum 4944* (US); **Potosi**, *M. Cárdenas 3733* (GH, US).

**4. *Bougainvillea berberidifolia*** Heimerl, Denkschr. Kaiserl. Akad. Wiss., Wien Math. -Naturwiss. Kl. 70: 121. 1900. Type: Bolivia, s. loc., *Cuming s.n.* (lectotype designated by Standley [8]: B).

*Bougainvillea berberidifolia* f. *oblongibracteata* Heimerl, Denkschr. Kaiserl. Akad. Wiss., Wien Math.-Naturwiss. Kl. 70: 121. 1900. Type: Bolivia, s. loc., *T. C.* *Bridges* *s.n.* (lectotype designated here: K [K000572706]).

*Bougainvillea berberidifolia* f. *cyclobracteata* Heimerl, Denkschr. Kaiserl. Akad. Wiss., Wien Math. -Naturwiss. Kl. 70: 121. 1900. Type: Bolivia, Pulquina and Comarapa, 1900 m., 1 April 1911, *T. Herzog 1799* (lectotype designated here: L [L1691803]; isolectotype: F [F0BN003090]).

Distribution: Bolivia, Paraguay.

Habitat: spiny thickets.

Specimens examined: BOLIVIA. **Saipina**, *T. Chen 20110122A* (USZ); **Santa Cruz**: Vallegrande, *G.A. Parada-Gutierrez et al. 2716* (MO, USZ); Caballero, *M. Nee*, *D. Villarroel & O. Colque 53749* (MO, US). PARAGUAY. **Nueva Asuncion**, Parque Nacional Teniente Enciso, *W. Hahn 1385* (SPF).

**5**. ***Bougainvillea campanulata*** Heimerl, Annuaire Conserv. Jard. Bot. Genève 17: 229 1913; et in Meded. Herb. Leid. No. 19: 33. 1913. Type: Bolivia, left bank of the Río Pilcomayo, 400 m, **Nov.** 1910, *T. C. J. Herzog 1124* (lectotype designated by Standley [8]: S [S07-13145].

*Bougainvillea herzogiana* Heimerl, Meded. Rijks-Herb. 27: 12. 1915. Type: Bolivia, Santa Cruz. Charakterstrauch im Dornbusch des Mte. Grande by Fortin Gurayus, Jun. 1907 *T. Herzog erste Reise no. 127* (holotype, L [L1692500]; isotype, MO [MO1432372]).

Distribution: Argentina, Bolivia, west-central Brazil, and Paraguay.

Habitat: ravines near rivers and basins.

Specimens examined: BOLIVIA. **Saipina**, *T. Chen* 20110122B (USZ); **Santa Cruz**: Manuel Maria Caballero, *M. Nee, D. Villarroel & O. Colque 53750* (MO, SPF, US). ARGENTINA. **Salta**, San Martin, A. *Charpin & U. Eskuche AC20483* (US). **Tucuman**: Trancas, S. *Venturi* 2128 (A, US); Burruyacu, *T. Stuckert 12356* (K). BRAZIL. **Mato Grosso do Sul**: Corumbá, *E. Pereira 154* (NY, RB). 

**6. *Bougainvillea infesta*** Griseb., Abh. Königl. Ges. Wiss. Göttingen 24: 40. 1879. Type: Argentina, Prov. Salta, Orán, an d. S. Seite d. Campo Grande, Oct. 1873, *P.G. Lorentz & G. Hieronymus 415* (holotype: GOET [GOET008952]; isotypes: B [B100715473], CORD [CORD00005765], GOET [GOET008953], K [K000494714]).

*Bougainvillea fasciculata* var. *spinosa* Brandão & Laca-Buendia, Daphne, 4(3):21–22., 1994. Type: Brazil. Minas Gerais, minicipio de São João da Ponte, estrada para Capitão Enéas, Caatinga/Mata Ciliar do Rio Verde, 16 Oct. 1991, *M. Brandão* *& J. P. Laca-Buendia* *21605* (holotype, PAMG [PAMG45000]). 

Distribution: Argentina, Bolivia, Brazil, Paraguay.

Habitat: dry forests.

Specimens examined: ARGENTINA. **Jujuy**: Esperanza, *R. E. Fries 524* (US); Ledesueva, *S. Venturi 5404* (US); San Pedro, S. *Venturi 5045* (US); **Salta**: Oran, Abra Grande, S. *Venturi 5507* (A (2 sheets), US); Oran, Cartagal, S. *Venturi 8683* (US). BOLIVIA. **Santa Cruz**: Cordillera, Boyuibe, *S. G. Beck & M. L. Beck 9398* (MO). BRAZIL. **Mato Grosso do Sul**: Ladário, *G. A. Damasceno Júnior 2820* (COR). PARAGUAY. **Nova Asuncion**: Parque Nacional Teniente Enciso, *W. Hahn 1678* (SPF).

**7. *Bougainvillea modesta*** Heimerl, Denkschr. Kaiserl. Akad. Wiss., Wien Math.-Naturwiss. Kl. 70: 118. 1901. Type: Bolivia, La Paz, near Coroico, *M. Bang 2398* (lectotype first-step designated by Standley [8] and second-step designated here: WU [WU0025182]; isolectotypes: B [B100715386], E [E00414164; E00414165], F, K [K000572703], M [M0274577], MICH [MICH1115347], MO [MO-934087], NY [NY00169740], US [US00931093; US00102985]).

*Bougainvillea fasciculata* Brandão, Anais XXXVII Congresso Nacional de Botânica. Ouro, 149–158. 1986, **syn. nov.** Type: Brazil. M. Brandão 2002 (Holotype: PAMG [PAMG1659]). 

Bougainvillea luteoalba Heimerl in Valenzuela., Guía Árbol. Bolivia 593. 1993, nom. invalid. (Art. 38.1 & 40.1), **syn. nov.**

Distribution: Bolivia and Brazil.

Habitat: in semi-deciduous dry forests.

Note: Standley [8] merged *B. modesta* with *B. praecox*. However, it can be easily distinguished from the latter by the straight or slightly constricted perianth tube. Heimerl [7] erroneously described *Bougainvillea modesta* to be a tree 25 m tall.

The perianth morphology, as well as the leaf shape (ovate to ovate-elliptic) and size (5–7 cm long, 3–4 cm wide), indicates that *B. luteoalba* and *B. fasciculata* are typical representatives of *B. modesta*, and therefore treated as new synonyms of the latter. The status of *B. luteoalba* is also supported by molecular data.

Specimens examined: BOLIVIA. **Beni**, *S.G. Beck 5976* (MO, LPB); **El Torno**, *T. Chen 2011012101C* (USZ); **Tarija**, *J. Pensiero & S. Marino 4467* (GH); **Trinidad**: Cercado, *E. Werdermann 2546* (LPB). BRAZIL. **Espírito Santo**: Santa Teresa. *V. Demuner 90* (RB).

**8**. ***Bougainvillea stipitata*** Griseb., Abh. Königl. Ges. Wiss. Göttingen 19: 88–89. 1874. ≡ *Bougainvillea stipitata* var. *grisebachiana* Heimerl. Denkschr. Kaiserl. Akad. Wiss., Wien Math.-Naturwiss. Kl. 70: 116. 1900, nom. invalid. (Art. 26.2). Type: Argentina, Prov. Córdoba, Vorbergen der Sierra bei Ascochinga, Apr. 1871, *P. G. Lorentz 374* (Holotype: GOET [GOET008958]); isotypes: B [B100715469], CORD [CORD00005752, CORD00005753].

*Bougainvillea frondosa* Griseb., Abh. Königl. Ges. Wiss. Göttingen 19: 89. 1874. Type: Argentina, Prov. Catamarca. Fuerte de Andalgalá, 14 Jan. 1872, *P. G. Lorentz 338* (lectotype designated here: GOTH [GOET008957]; isolectotypes: CORD [CORD00005757, CORD00005758]).

*Bougainvillea trollii* Heimerl, Notizbl. Bot. Gart. Berlin-Dahlem 11: 464. 1932. Type: Bolivia. Alto de Santa Rosa—Carapari. Sommergr[üner] Wald, 6 Oct. 1927, *C. Troll 379* (holotype: B [B100715467]; isotype: M [M0274580]), **syn. nov.**.

*Bougainvillea longispinosa* Rusby, Mem. Torrey Bot. Club 6: 109. 1896. ≡ *Bougainvillea stipitata var. longispinosa* (Rusby) Heimerl, Denkschr. Kaiserl. Akad. Wiss., Wien. Math.-Naturwiss. Kl. 70: 116. 1901. Type: Bolivia, Turedon, vic. Cochabamba, 1891, *M. Bang 1123* (lectotype first-step designated by Standley [8] and second step designated here: B [B100715474]; isolectotypes: BR [BR0000009166790], E [E00414166], GH [GH 00970002, GH00037344], K [K000572705], NY [NY00342025, NY00342026], US [US00623571, US00102984], BR [BR0000009166790]).

*Bougainvillea stipitata* var. *fiebrigii* Heimerl. Bot. Jahrb. Syst. 42: 76. 1908, “Fiebrigii”. Type: Bolivia, Tarija, Paicho W. Tarija, 3000 m, 5 Feb. 1904, *K. Fiebrig 3049* (lectotype designated first-step by Standley [8] and second step designated here: B [B100715382]; isolectotypes: A [A00970003] B [B100715383], JE [JE00013008], NY [NY00658984], S [S07-13153], US [US00623570]).

*Bougainvillea stipitata* var. *kuntzeana* Heimerl., Denkschr. Kaiserl. Akad. Wiss., Wien Math.-Naturwiss. Kl. 70: 117. 1900. Type: Bolivia, Tunari, 1500 m, April 1892, *C.E.O. Kuntze s.n.* (lectotype first-step designated by Standley [8] and second step designated here: B [B100715475]; isolectotypes:, NY [NY00658982, NY00658983]). *Bougainvillea stipitata* Griseb. var. *stuckertiana* Heimerl, Annuaire Conserv. Jard. Bot. Genève 17: 228. 1913. Type: Argentina, Córdoba, San Alberto, Tránsito, 5 dec. 1905, *T. J. V. Stuckert 10316* (lectotype designated here: CORD [CORD00002627]).

Distribution: Northeastern and northwestern Argentina, Bolivia, southern and west-central Brazil.

Habitat: Associated with watercourses, up to 3000 m asl.

Note: The thorn structure and leaf shape used to differentiate varieties of *Bougainvillea stipitata* are of no principal systematic value. They are not the type of feature that can be used on their own to characterize taxa of *Bougainvillea*.

*Bougainvillea trollii* has greenish, ovate to ovate-elliptic bracts and salver-shaped (constricted in middle) flowers similar to *B. stipitata*. Their morphological features are more or less in the range of continuous variation. They also overlap in their distribution. *B. trollii* is therefore treated as a new synonym of *B. stipitata*.

Specimens examined: ARGENTINA. **Catamarca**: Andalgalá, *P. Jörgensen* 1092 (GH, US); Fuerta de Andalgala, *F. Schickendantz 29* (K); *R. Pearce s.n.* (K); Sal Geiubó, S. Venturi 2702 (A, GH, K); **Córdoba**, *Hieronymus* 357 (K); **Jujuy**: Arroyo del Medio, *R. E. Fries 359* (US); Ledesma, *S. Venturi 5412* (US); **Salta**, *A. Krapovickas 40302* (F); Capital, San Bernardo, *B. Sparre 1149* (K); Coronel Moldes, H. H. *Bartlett 19716* (GH, US); Estancia La Despensa, La Caldera, *A. T. Hunziker 1632* (GH, US); Guadupa, Alemania, *S. Venturi 9806* (GH, US); Merlo, *A. Burkart 13946* (K); Rosario de la Frontera, Los Baños, *S. Venturi 9321* (GH, US); **Tucuman**: Capital, Barranca Colorada, *S. Venturi 822* (A, US). BOLIVIA. **Bermejo**, *K. Fiebrig 2352* (GH, US); **Chuquisaca**: *K. Fiebrig 2689* (A, F, US). **Cochabamba**: Ayopaya, *M. Cárdenas 4294* (US); Mizque, *W.J. Eyerdam 25344* (K); **Cordillera**, Between Charagua and Eytí, *M. Cárdenas 4750* (US); **Gran Chaco**: Tatarenda, *R. E. Fries 1478* (US); **Florida**, Andrés Ibáñez, *M. Nee 55705* (MO); *D. Villarroel, M. Vargas C. & F. Seidel 441* (MO, USZ); *L. Arroyo et al. 3321* (L, MO, USZ); **Santa Cruz**, *C. Davidson 5123* (F); Caballero, *M. Nee, M. Sundue & A. Carrasco 52182* (MO, US); *Caballero*, *R. K. Brummitt, J. R. I. Wood & D. C. Wasshausen 19237* (US); Cordillera, M. *Nee 53157* (MO, US); Vallegrande, *G. A. Parada & V.D. Rojas 2657* (L, MO, USZ); Vallegrande, *M. Nee & J.M. Mendoza* 57603 (MO, USZ, W]).

**9. *Bougainvillea praecox*** Griseb., Abh. Königl. Ges. Wiss. Göttingen 24: 40. 1879. Type: Argentina, Prov. Salta. Dragones, Aug. 1873, *P.G. Lorentz & G. Hieronymus 611* (holotype: GOET [GOET008959]; isotype: B [B 10 0715476], CORD [CORD00005768]).

*Bougainvillea malmeana* Heimerl, Denkschr. Kaiserl. Akad. Wiss., Wien. Math.-Naturwiss. Kl. 70: 119, pl. 1, Figure 1 (1900). Type: Brazil, prov. Matto Grosso, Corumba, 18 Aug. 1894, *G. O. A. Malme 1772* (lectotype designated here: B [B100715471], isolectotypes: GH [GH 00037345], US [US1194802]). 

*Bougainvillea praecox* var. *rhombifolia* Heimerl, Verh. K.K. Zool.-Bot. Ges. Wien 62: 4. 1912. Type: Bolivia, between Ipwassu & Fortin d’ Orbigny, 8 **Nov.** 1910, *T. Herzog 1073* (lectotype designated here: L [L.1692509]; isolectotype: MO [MO100182597]).

*Bougainvillea praecox* var. *spinosa* Chodat & Hassl., Bull. Herb. Boissier, sér. 2, 3: 415. 1903. Type: Paraguay, Iter ad Paraguariam Septentrionalem. Prope Conception, *E. Hassler 7414* (lectotype designated here: P [P00712544], isolectotypes: BM [BM000098919], MPU [MPU018936], NY [NY00579251], P [P00753858], S [S07-13149], UC [UC934869]).

Distribution: Northeastern and northwestern Argentina, Bolivia, west-central Brazil, Paraguay.

Habitat: near riverbanks.

Note: *Bougainvillea praecox* has striking similarities to *B. modesta* [8], particularly in the color and shape of the bracts. However, the shape of the perianth tube is quite distinct from *B. modesta* and comparable to the tube of cultivated species. The cylindrical perianth is constricted in the middle (or slightly above the middle) and the basal portion is enlarged and ellipsoid.

Specimens examined: BOLIVIA. **Beni**: The Amazon Basin, *O. E. White 973* (GH, US); Between Ipawassu & Fortin d’Orbigny, *T. Herzog 1076* (MO). **Santa Cruz**: ca. 73 km W of Samaipata on Carretera Fundamental 4, *C. Davidson 3829* (RSA). BRAZIL. **Mato Grosso**: Corumba, *G. O. A. Malme s. n.* (S); **Nova Odesa**, Jardim Botanico Plantarum, *T. Chen 2012063001* (HPL).

**10. *Bougainvillea spectabilis*** Willd., Sp. Pl. 2(1): 348. 1799, nom. cons. prop. ≡ *Bougainvillea bracteata* Pers., **Syn.** Pl. 1: 418. 1805, nom, illeg. (Art. 52.1), “*Bugainvillaea*”. ≡ *Tricycla spectabilis* (Willd.) Poir., Encycl., Suppl. 5: 359. 1817. ≡ *Bougainvillea spectabilis* var. *typica* Heimerl. Bot. Jahrb. Syst. 21: 623. 1896, nom. invalid. (Art. 24.3). Type: [icon] Lamarck, Tabl. Encycl. 2: t. 293. 1792, upper part (holotype); Brazil, Rio de Janeiro, July 1767, *P. Commerson s.n.* (epitype designated by Lack [1]: P [P00169376]; isoepitypes: G [G00341746, G00341747], MPU [MPU018937], P [00307018, P00499733, P00672131, P01903639]).

*Bougainvillea brasiliensis* J. F. Gmel, Syst. Nat. 2: 632. 1791, nom. rej. prop. Type: Brazil, Rio de Janeiro, “in circa Rio-Jan. in vicinis nemoribus,” July 1767, *Commerson 192* (neotype designated by Chagas & Costa-Lima [24]: P [P00307018]; isoneotypes: G [G00341746, G00341747], MPU [MPU018937], P [P00499733, P00672131, P01903639]).

*Josepha augusta* Vell., Fl. Flumin.: 154. 1829. Type: Brazil, “prope Xistos,” *Velloso s.n.* (holotype: R, destroyed); [icon] drawing by Frei Francisco Solano, before 1791 (lectotype designated by Lack [1]: R).

*Bougainvillea virescens* Choisy, Prodr. 13(2): 437. 1849. ≡ *Bougainvillea spectabilis* var. *virescens* (Choisy) J. A. Schmidt, Fl. Bras. 14(2): 351. 1872. Type: Brazil, 30 Nov. 1834. P. W. Lund 393 (lectootype designated here: G-DC [G00689828])

*Bougainvillea speciosa* Schnizl., Iconogr. Fam. Regn. Veg. 2: t. 104. 1850. Type: [icon] Schnizl., Iconogr. Fam. Regn. Veg. 2: t. 104, Figures 21–26. 1850 (lectotype designated here).

*Bougainvillearubriflora* Brandão, Anais XXXVII Congr. Nac. Botânica. Ouro, 151. 1986, **syn. nov.** Type: Brazil, Jaiba, strata Jaiba-Matia Gardosa, *M. Brandao 3609* (holotype: PMAG [ PMAG4264]).

*Bougainvillea spectabilis* var. *hirsutissima* J. A. Schmidt, Fl. Bras. 14(2): 351. 1872. Type: Brazil, Rio de Janeiro, *H. W. Schott 5565* (not located).

*Bougainvillea spectabilis* var. *parviflora* Mart. ex J.A. Schmidt., Fl. Bras. 14(2): 351. 1872. Type: Brazil, *C. F. P. Martius 64* (lectotype designated here: P [ P00712545]; isolectotypes: GH [GH 00969999; GH 00970000], P [P00712546, P00712547]).

Distribution: Native to Brazil; cultivated mostly in the tropics and subtropics.

Habitat: forest edges, coastal thickets, dry forests, disturbed sites near villages, gardens, parks, along roadsides from near sea level to ~1000 m.

Specimens examined: BRAZIL. **Amazonas**: Ad Ega, *L. Riedel s.n.* (NY); **Bahia**, Vicinity of Joazeiro, *J. N. Rose & P. G. Russell 19780* (NY); **Ceará**: Fortaleza, *F. E. Drouet 2602* (K, NY); **Minas Gerais**: Ilheu, *Y. E. J. Mexia 4991* (A, K, NY); Lambari, *E. Pereira 10703* (K); Tombos Serra dos Quintinhos, *C. Martins 39679* (K); **Rio de Janeiro**, *J. Miers 3107* (K); *A. Glaziou 18421* (K, NY); *G. Gardner 103* (GH); Pr. Barra de Sao Joao, *G. F. J. Pabst 7014* (K, NY); Base of Dois Irmaos, *J. N. Rose & P. G. Russell 20238* (NY); Environs de Rio de Janeiro, *A. Glaziou 12113* (K); **St**. **Paul and Rio**, *J. Weir 26* (K).

**11. *Bougainvillea glabra*** Choisy, Prodr. 13(2): 437. 1849. ≡ *Bougainvillea spectabilis* var. *glabra* (Choisy) Hook., Bot. Mag. 80: 4811. 1854. ≡ *Bougainvillea glabra* var. *typica* Heimerl., Denkschr. Kaiserl. Akad. Wiss., Wien. Math.-Naturwiss. Kl. 70: 111. 1900, nom. invalid (Art. 24.2). Type: Brazil. Rio de Janeiro, *Gaudichaud* 423 (lectotype designated by Kellogg [25]: G-DC [G00074302]; isotypes, P [P00712532, P00712533]).

*Bougainvillea pomacea* Choisy, Prodr. 13(2): 438. 1849. ≡ *Bougainvillea glabra* var. *pomacea* (Choisy) Luetzelb., Estud. Bot. Nordéste 3: 29. 1923. Type: Brazil, Bahia, Serra de Jacobina, montibus de la Jaxobina ad Bahiam, 1836, *S. J. Blanchet 2573* (lectotype designated here: P [P00712541]; Isolectotypes: BR [BR0000005230686, BR0000005231010], MEL [MEL2444672], NY [NY00342027], P [P00712542, P00712543]).

*Bougainvillea brachycarpa* Heimerl, Bot. Jahrb. Syst. 11: 88. 1889. ≡ *Bougainvillea glabra* var. *brachycarpa* (Heimerl) Heimerl, Denkschr. Kaiserl. Akad. Wiss., W ien. Math.-Naturwiss. Kl. 70: 113. 1900. Type: Brazil, *F. Sellow 627* (holotype: B [B 10 0715388]).

*Bougainvillea glabra* var. *acutibracteata* Heimerl, Vidensk. Meddel. Naturhist. Foren. Kjøbenhavn 1890: 159. 1891. Type: Brazil, Rio de Janeiro, 28 Nov. 1878, *A. Glaziou 11417* (lectotype designated here: P [P04999897]; isolectotype, K [K000572709]).

*Bougainvillea glabra* var. *graciliflora* Heimerl, Denkschr. Kaiserl. Akad. Wiss., Wien. Math.-Naturwiss. Kl. 70: 112. 1900. Type: Brazil, St. Catharina, *F. Sellow 5597* (lectotype designated here: B [B100715478]).

Distribution: Native to Brazil. Cultivated mostly in the tropics and subtropics.

Habitat: In well-drained sandy soils, slopes, mesas, and disturbed rocky soils; from sea level to 1000 m.

Specimens examined: BRAZIL. **Bahia**, 26 km de Maracás rumo Tambril na decida da Serra, *E. Pereira 9717* (K, NY); Jacobina, *L. Coradin 6183* (NY); Ilhéus, *M. B. M. da Cruz 0001* (NY); Alguns km de Jequié, *E.P. Heringer 12819* (NY); **Espírito Santo**: Linhares, Margem do Rio Doce, *R. P. Belém 1580* (NY [NY642320]); **Minas Gerais**: Ponto dos Volantes, *G. Eiten & L.T. Eiten 10896* (NY); **Santa Catarina**: Orleans, *R. Reitz & Klein 1759* (NY).

**11a. *Bougainvillea glabra*** var. ***obtusibracteata*** Heimerl, Vidensk. Meddel. Naturhist. Foren. Kjøbenhavn 1890: 158. 1891. Type: Brazil, Rio de Janeiro, Novo Friburgo, 22 June 1880, *A. Glaziou 12112*, (lectotype designated here: B [B100715384]); isolectotypes, P [P00712529, P00712530, P00712531], K [K001138189].

*Bougainvillea arborea* Glaz., Mém. Soc. Bot. France 3: 561. 1911, **syn. nov.** Type: Brazil, Rio de Janeiro, Novo Friburgo, *A. Glaziou 4177* (lectotype designated here: P [P00712525]; isolectotypes: K [K000572708], P [P00712526]).

Distribution: Native to northeastern, southern, and southeastern Brazil. Occasionally cultivated in the tropics and subtropics.

Habitat: frequent on forested slopes.

Note: Most herbarium specimens collected in Brazil and identified by A. Glaziou as *B. arborea* have obtuse bracts and the base of the perianth tube obturbinoid, which are features consistent with the morphology of *B. glabra* var. *obtusibracteata*. In 1916, Heimerl even noted “*B. aborea*” on the label of *A. Glaziou 12112*, which is the type of *B. glabra* var. *obtusibracteata*. Therefore, we treat *B. aborea* as a new synonym here.

Specimen examined: BRAZIL. **Parana**, *P. K. H. Dusén 16772* (GH). CHINA. **Shenzhen**, Fairy Lake Botanical Garden (from USA. **Miami**, Fairchild Tropical Botanical Garden, originally from Brazil), *T. Chen 2020031202* (SZG).

### 3.2. Cultivated Hybrid

*Bougainvillea* × *buttiana* Holttum & Standl. ≡ *Bougainvillea buttiana* Holttum & Standley, Publ. Field Mus. Nat. Hist., Bot. Ser. 23(2): 44. 1944. Type: Singapore, cultivated in Singapore Botanical Garden as *Bougainvillea* ‘Mrs. Butt,’ purchased from L. R. Russell, Richmond, Illinois, in 1923, 1 July 1938, *R. E. Holttum s.n.* (holotype: F).

*Bougainvillea* × *buttiana* Holttum & Standl. was named as a new species based on a plant that had been cultivated in the Singapore Botanical Garden [26]. It was originally from a garden in Cartagena, Colombia, and taken to Trinidad in 1910 as a cultivar ‘Mrs. Butt’. It was presumed by Gillis [27] to be a hybrid between *B. peruviana* and *B. glabra*.

Cultivated varieties are believed to be from crosses mainly involving *B. glabra*, *B. peruviana*, and *B. spectabilis*, which have larger and more colorful bracts than other species. *Bougainvillea* cultivars with large bracts of various colors have tremendous promise in horticulture. Hundreds of cultivars have been produced by physical, chemical, and biological means for cultivation in tropical and subtropical regions around the world [28,29]. The names of cultivated varieties, such as *Bougainvillea glabra* var. *sanderiana* Dimmock, *Bougainvillea glabra* var. *alba* Mendes & Viégas, etc., are being verified and will be treated separately.

## 4. Materials and Methods

### 4.1. Sample Collection and DNA Extraction

Leaf samples of *Bougainvillea* utilized in this research were collected in South America (Argentina, Bolivia, Brazil, Ecuador, Peru), China, and the United Kingdom (Table S3). Samples were obtained from either silica gel dried leaves or herbarium specimens. The modified CTAB method [30] was used to extract total genomic DNA from the leaf samples. DNA quality was assessed through agarose gel electrophoresis, nanodrop, and Qubit 2.0.

### 4.2. Sequencing, Assembly, and Annotation of Bougainvillea Plastid Genomes

DNA was fragmented using the Covaris ultrasonic disruptor, after which short insert sized (350–400 bp) libraries were prepared according to the manufacturer’s protocol of the Nextera XT DNA Library Preparation Kit. Paired-end sequencing (2 × 150 bp) was conducted in Illumina Novseq 600 platform and approximately 10.0 GB of raw data were generated for each sample. Sequencing depths ranged from 263.8× to 1627.3×. The adaptor sequences, undersized inserts, duplicated reads, and poor-quality reads were filtered out using the NGS-QC toolkit [31].

The high-quality reads obtained were initially spliced in SPAdes 3.11.0–St. Petersburg genome assembler [32]. Using the default parameters (without the cutoff parameter), all the scaffolds that could be assembled from the clean data were spliced together. Then BlastN was performed with the published genome of *Bougainvillea spectabilis*. The comparison threshold was set to e-value 1 × 10^−10^ and protein similarity threshold of 70%. The scaffolds that matched the genes were selected and the splicing coverage was sorted. The fragments with low coverage, which were obviously not included in the target genome, were removed. Subsequently, the collected target fragment sequences were extended and merged using PRICE software [33] to minimize the number of scaffolds. The number of iterations was set to 50. The results of iterative splicing were aligned to the original sequencing reads using Bowtie 2 [34]. The matched pairs of reads were then selected and re-spliced using SPAdes 3.11.0 [32].

Plann software [35], cpGAVAS [36], and DOGMA [37] were used to annotate the assembled chloroplast genomes. Protein-coding gene annotation was confirmed through BlastN searches, while rRNA and tRNA annotations were verified using RNAmmer 1.2 [38] and tRNAscan-SE v2.0 [39]. After annotation, the circular genome maps were constructed using the OGDRAW v1.3.1 [40]. The cpDNA sequences with their accession numbers were deposited in NCBI GenBank (Table S4).

### 4.3. SNPs and Indels Analysis

The variation among the plastid genomes of *Bougainvillea* was analyzed through SNPs (Single Nucleotide Polymorphisms) and indels (insertions and deletions) identification. SNPs and indels were identified in MUMmer 4 [19] and Geneious Prime 2020.2 [20] using *B. glabra* as the reference genome. The nonsynonymous (Ka) and synonymous (Ks) substitution rates of the protein-coding genes were also determined through the Selecton 2007 program [41].

### 4.4. Phylogenetic Analysis

To elucidate the phylogenetic relationships within *Bougainvillea*, 11 newly sequenced plastid genomes were included in the analysis, along with the eight sequences of *Bougainvillea* from previous studies, and six additional sequences of Nyctaginaceae from GenBank (Table S4). Four chloroplast genomes from the allied family Petiveriaceae were also used as outgroups. From these datasets, 79 protein-coding sequences were extracted and aligned using MAFFT v7.388 [42] software embedded in Geneious Prime 2020.2 [20]. When necessary, alignments were manually adjusted to remove ambiguous areas.

After the alignment, Maximum Likelihood (ML) analysis was carried out in RAxML 8.2.11 using the GTR+I+G nucleotide substitution model [43,44]. The appropriate model was determined through jmodeltest2 performed in CIPRES Gateway [45]. The consensus tree was inferred from 1000 replicates using *Seguieria aculeata*, *Rivina humilis*, *Petiveria alliacea*, and *Monococcus echinophorus* as outgroups. In addition, Bayesian Inference (BI) was also analyzed using MrBayes 3.2.6 with the general time-reversible model of DNA substitution and a gamma distribution rate variation across sites [46]. BI analyses were conducted in CIPRES Gateway [45] with the setting of four MCMCs running for one million generations with sampling every 1000 generations, and the first 25% discarded as burn-in. Branches with ML bootstrap support above 75 and Bayesian posterior probabilities (BPP) above 0.95 were regarded as significantly supported.

### 4.5. Morphological Analysis

Digital images of specimens of *Bougainvillea* from various herbaria (A, B, BR, CORD, E, F, GH, GOET, K, LPB, MA, MICH, MO, MPU, NY, P, S, US, USZ, W) were used in the morphological study. In addition, fresh specimens from the living collection of *Bougainvillea* in the Shenzhen Fairy Lake Botanical Garden were used for morphological observations and floral dissections. Photographs of additional species of *Bougainvillea* were also retrieved from the Flora of Argentina [22] and Flora of the World Online [23]. The terminologies used to describe the materials were mainly based on the Kew plant glossary [47]. The table of diagnostic characteristics (Table S5) was based primarily on direct observations, but published descriptions and protologues were also used to complete the table.

## Data Availability

The complete chloroplast genome sequences of *Bougainvillea* samples used in the study were deposited in the NCBI GenBank under accession numbers OM044392- OM044400, MW123899-MW123903.

## Supplementary Materials

The following supporting information can be downloaded at: https://www.mdpi.com/article/10.3390/plants11131700/s1, Table S1: Genome features of *Bougainvillea* chloroplast genomes; Table S2: List of genes encoded by *Bougainvillea* chloroplast genomes; Table S3: *Bougainvillea* samples included in the study; Table S4: Data set included in the phylogenetic and comparative analyses of complete chloroplast DNA sequences (*Bougainvillea*, Nyctaginaceae); Table S5: Morphological comparison of *Bougainvillea* species; Figure S1: Insertion-Deletions (Indels) in the protein-coding genes of *Bougainvillea* chloroplast genomes.
