# Supplementary material for: Phylogeny and Taxonomic Synopsis of the Genus Bougainvillea (Nyctaginaceae)
Source: Plants (Basel). 2022 Jun 27;11(13):1700. doi: 10.3390/plants11131700 (PMC9269543; doi:10.3390/plants11131700)
Supplement: Supplementary file 1 [file plants-11-01700-s001.zip › Figure S1.pdf]

Figure S1. Insertion-Deletions (InDels) in the protein-coding genes of *Bougainvillea* chloroplast genomes

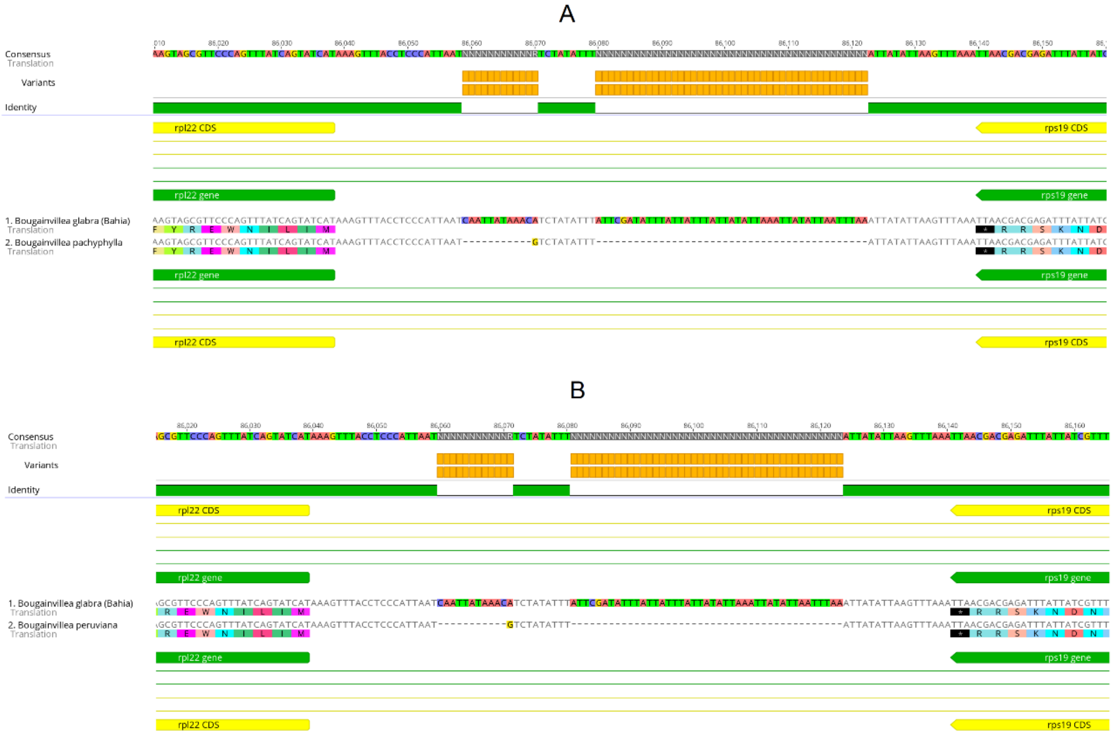

A large deletion in the *rpl22*-*rps19* intergenic spacer of (A) *B. pachyphylla* and (B) *B. peruviana*.

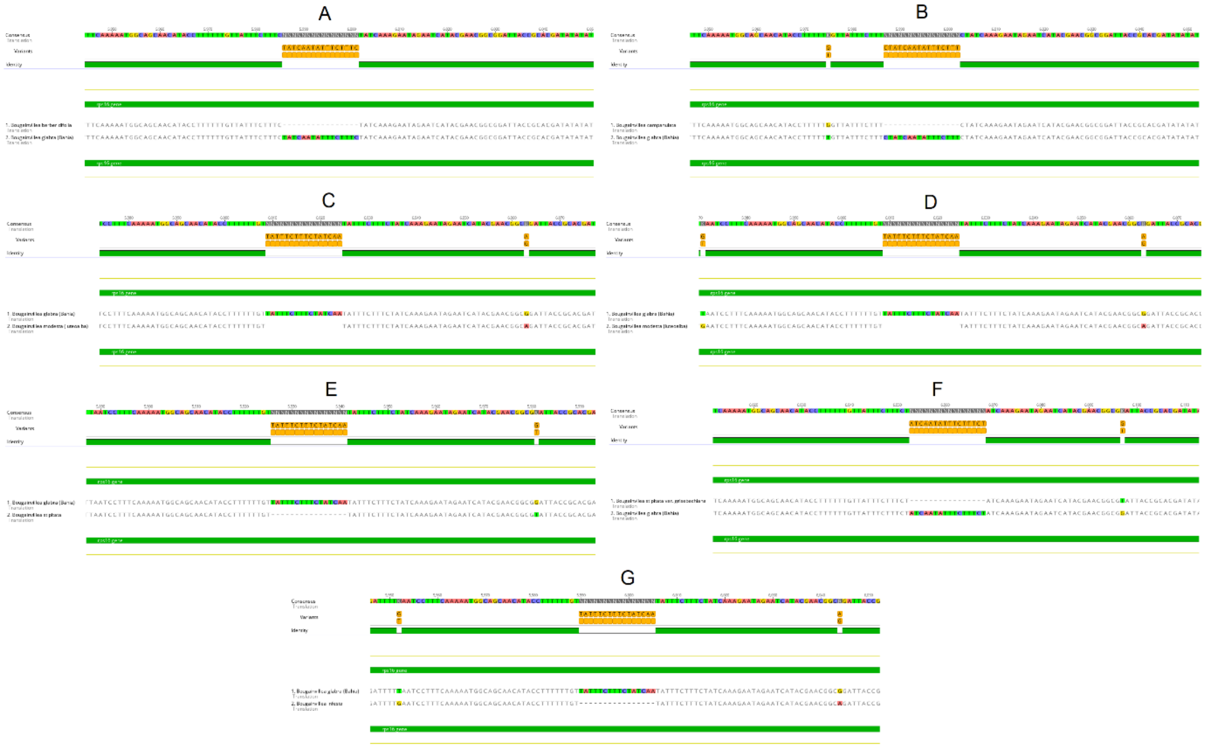

Deletion in the *rps16* intron of wild *Bougainvillea* species (A) *B. berberidifolia*, (B) *B. campanulata*, (C) *B. luteoalba*, (D) *B. modesta*, (E) *B. stipitata*, and (F*) B. stipitata* var. *grisebachiana*.

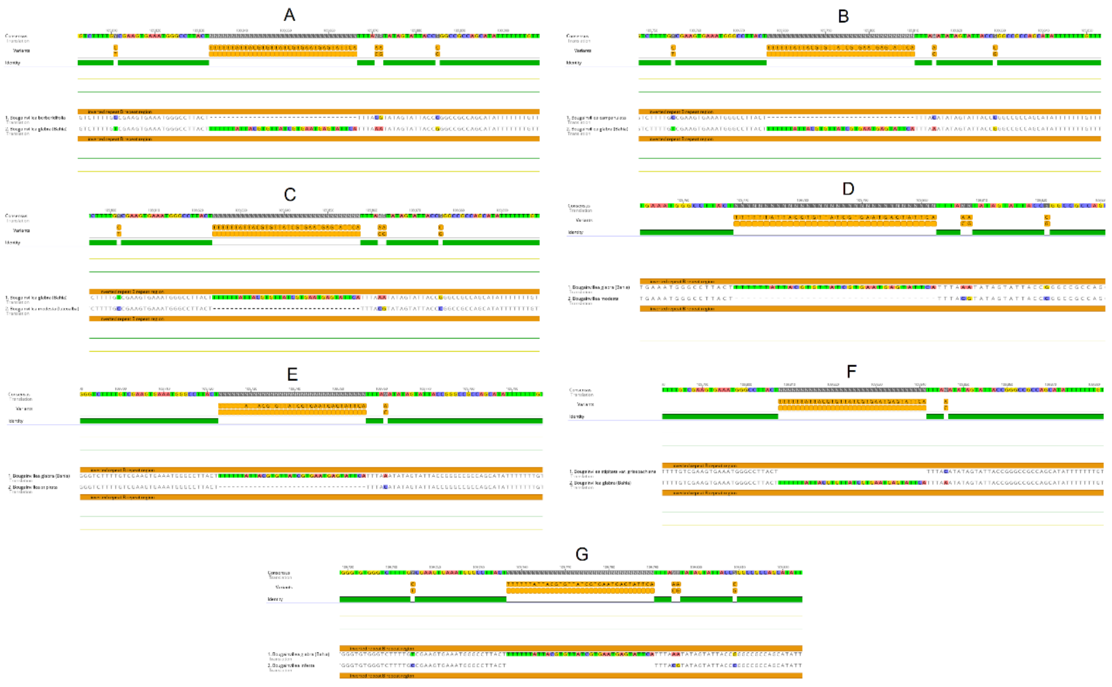

A large deletion in the *trnR-AGC-trnN-GUU* intergenic spacer of wild *Bougainvillea* species (A) *B. berberidifolia*, (B) *B. campanulata*, (C) *B. luteoalba*, (D) *B. modesta*, (E) *B. stipitata*, and (F*) B. stipitata* var. *grisebachiana*.

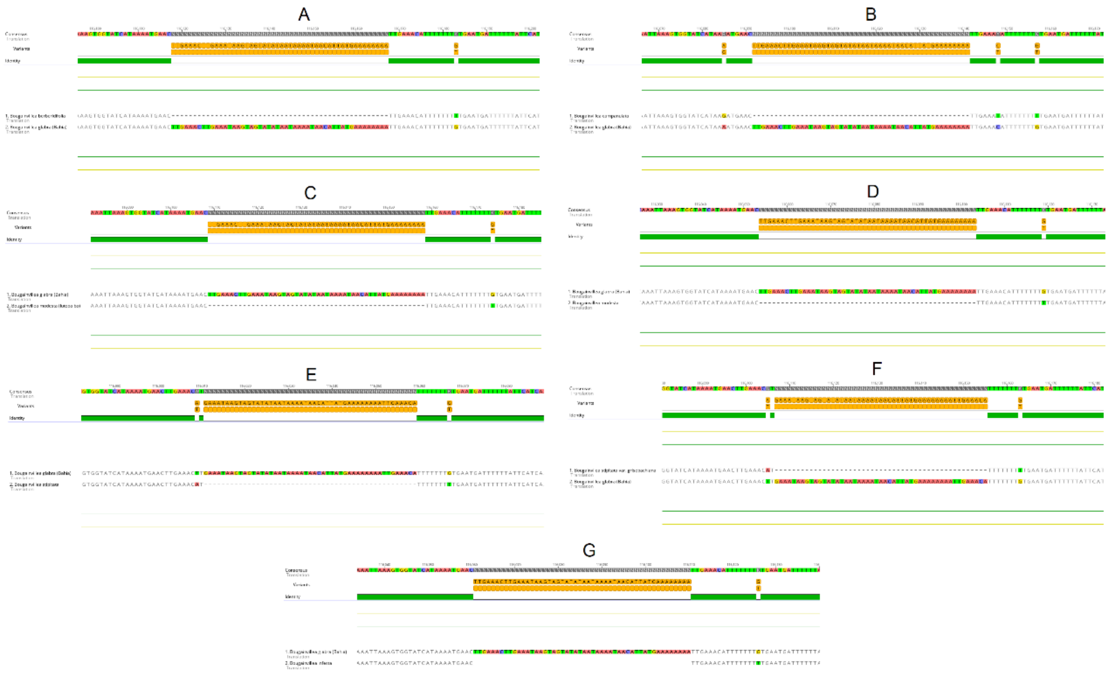

A large deletion in the *rpl32-trnL-UAG* intergenic spacer of wild *Bougainvillea* species (A) *B. berberidifolia*, (B) *B. campanulata*, (C) *B. luteoalba*, (D) *B. modesta*, (E) *B. stipitata*, and (F) *B. stipitata* var. *grisebachiana*.

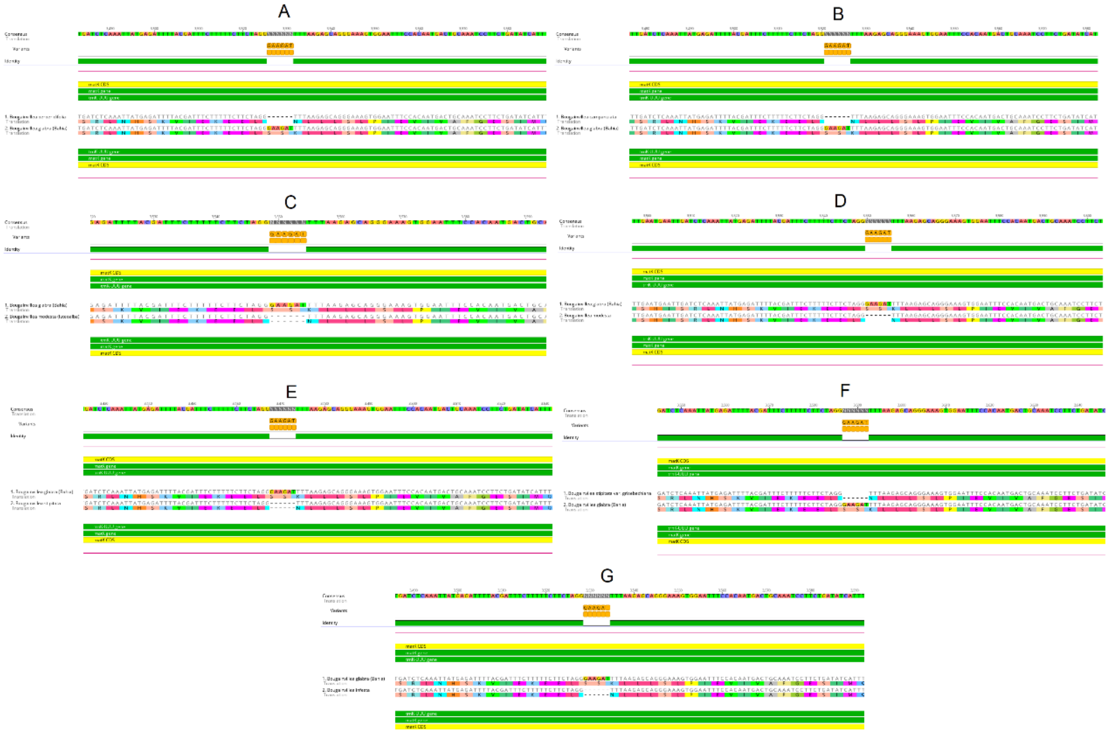

A small deletion in the *matK* gene of wild *Bougainvillea* species *(A) B. berberidifolia, (B) B. campanulata, (C) B. luteoalba, (D) B. modesta, (E) B. stipitata, (F) B. stipitata* var. *grisebachiana.*

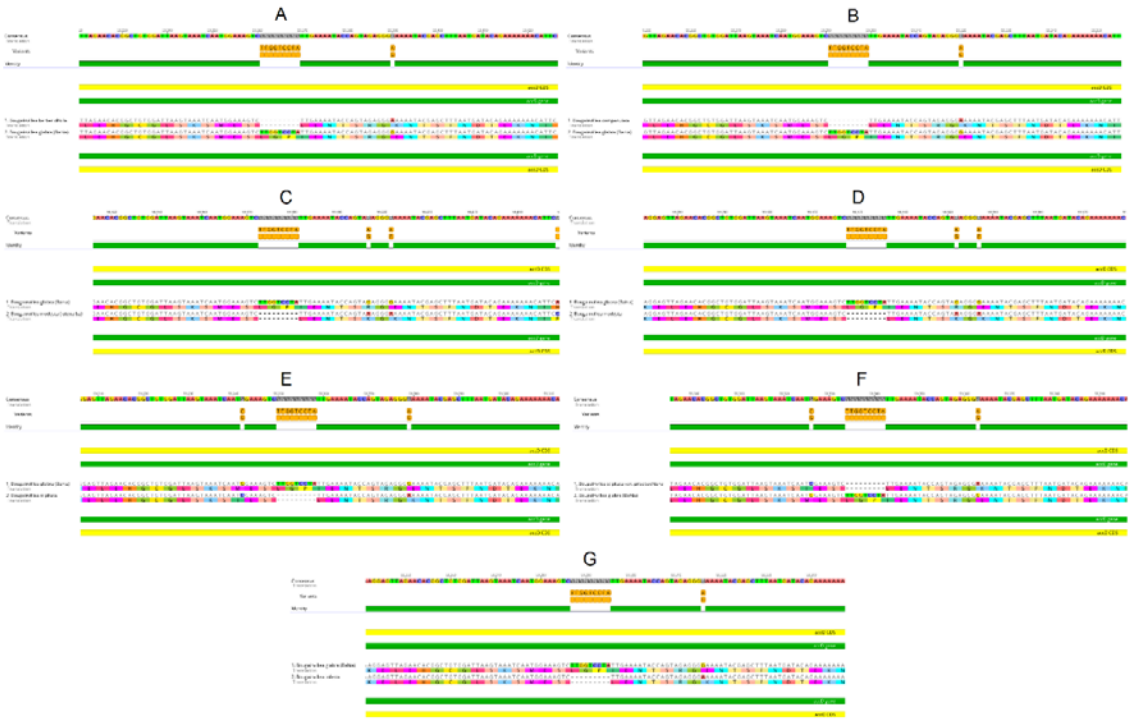

A small deletion (9 bp) in the *accD* gene of wild *Bougainvillea* species *(A) B. berberidifolia*, *(B) B. campanulata*, *(C) B. luteoalba*, *(D) B. modesta*, *(E) B. stipitata*, *(F) B. stipitata* var. *grisebachiana.*

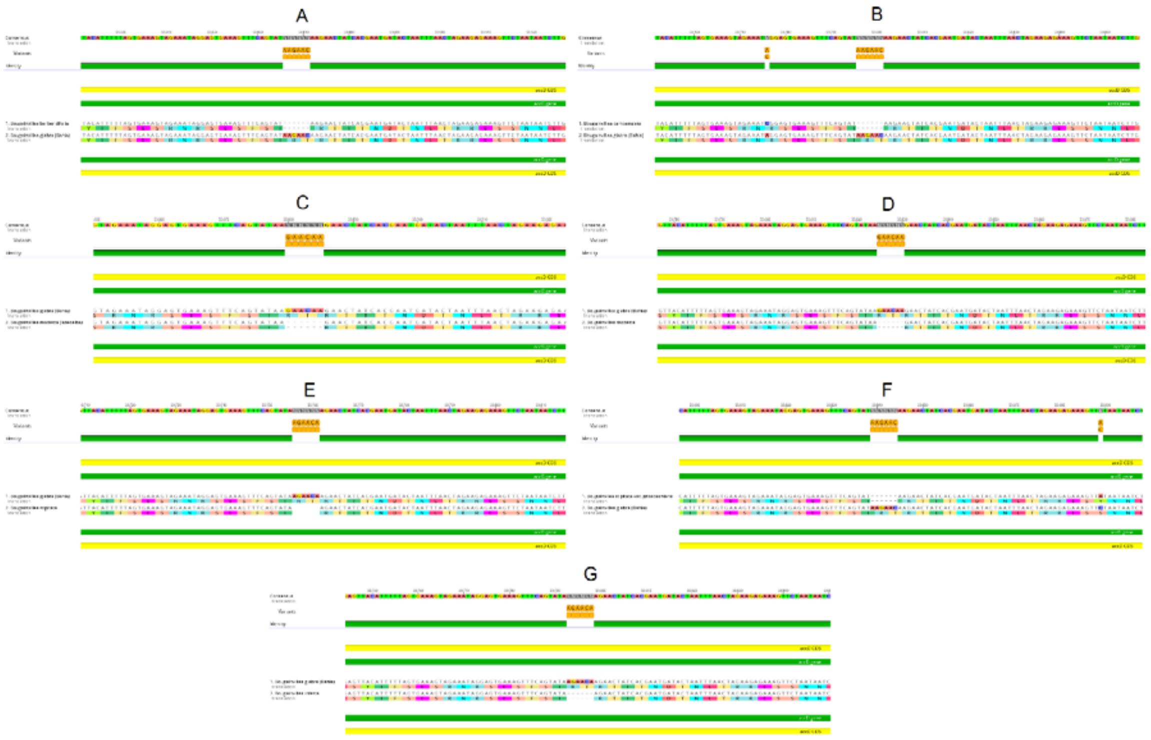

A small deletion (6 bp) in the *accD* gene of wild *Bougainvillea* species *(A) B. berberidifolia*, *(B) B. campanulata*, *(C) B. luteoalba*, *(D) B. modesta*, *(E) B. stipitata*, *(F) B. stipitata* var. *grisebachiana.*
