# Supplementary material for: Phylogeny and Taxonomic Synopsis of the Genus Bougainvillea (Nyctaginaceae)
Source: Plants (Basel). 2022 Jun 27;11(13):1700. doi: 10.3390/plants11131700 (PMC9269543; doi:10.3390/plants11131700)
Supplement: Supplementary file 1 [file plants-11-01700-s001.zip › Table S1.pdf]

Table S1. Genome features of *Bougainvillea* chloroplast genomes.

Species / cultivar	Genome size (bp)	LSC (bp)	SSC (bp)	IRs (bp)	Number of Genes				GC Content (%)			
					Total	CDS	tRNA	rRNA	Total	LSC	SSC	IRs
B. glabra	154,536	85,708	18,038	25,395	131	86	37	8	36.5	34.2	29.5	42.8
B. peruviana	153,966	85,159	18,025	25,391	131	86	37	8	36.6	34.3	29.6	42.8
B. pachyphylla	154,062	85,181	18,027	25,427	131	86	37	8	36.5	34.3	29.6	42.8
B. praecox	154,306	85,474	18,014	25,409	131	86	37	8	36.5	34.3	29.5	42.8
B. cultivar	154,520	85,688	18,078	25,377	131	86	37	8	36.5	34.2	29.5	42.8
B. spectabilis	154,541	85,694	18,077	25,385	131	86	37	8	36.4	34.2	29.5	42.7
B. arborea	154,828	85,958	18,028	25,421	131	86	37	8	36.4	34.1	29.5	42.8
B. berberidifolia	154,561	85,789	18,000	25,386	131	86	37	8	36.5	34.2	29.4	42.8
B. campanulata	154,529	85,700	18,059	25,385	131	86	37	8	36.5	34.2	29.4	42.8
B. infesta	154,385	85,596	18,017	25,386	131	86	37	8	36.5	34.3	29.4	42.8
B. modesta	154,387	85,618	17,997	25,386	131	86	37	8	36.5	34.3	29.5	42.8
B. luteoalba	154,457	85,687	17,998	25,386	131	86	37	8	36.5	34.2	29.5	42.8
B. spinosa	154,872	85,846	18,020	25,503	131	86	37	8	36.4	34.1	29.4	42.8
B. stipitata	154,667	85,861	18,036	25,385	131	86	37	8	36.5	34.2	29.4	42.9
B. stipitata var. *grisebachiana*	154,679	85,894	18,015	25,385	131	86	37	8	36.5	34.2	29.4	42.8
