# Supplementary material for: Phylogeny and Taxonomic Synopsis of the Genus Bougainvillea (Nyctaginaceae)
Source: Plants (Basel). 2022 Jun 27;11(13):1700. doi: 10.3390/plants11131700 (PMC9269543; doi:10.3390/plants11131700)
Supplement: Supplementary file 1 [file plants-11-01700-s001.zip › Table S2.pdf]

Table S2. List of genes encoded by _Bougainvillea_ chloroplast genomes

Gene category	Gene names
ATP Synthase	_atp_A, _atp_B, _atp_E, _atp_F*, _atp_H, _atp_I
NADH dehydrogenase	_ndh_A*, _ndh_B(X2)*, _ndh_C, _ndh_D, _ndh_E, _ndh_F, _ndh_G, _ndh_H, _ndh_I, _ndh_J, _ndh_K
Cytochrome b/f complex	_pet_A, _pet_B*, _pet_D*, _pet_G, _pet_L, _pet_N
Photosystem I	_psa_A, _psa_B, _psa_C, _psa_I, _psa_J
Photosystem II	_psb_A, _psb_B, _psb_C, _psb_D, _psb_E, _psb_F, _psb_H, _psb_I, _psb_J, _psbK_, _psb_L, _psb_M, _psb_N, _psb_T, _psb_Z
RubisCO large subunit	_rbc_L
Ribosomal protein genes (large subunits)	_rpl_2(x2)*, _rpl_14, _rpl_16*, _rpl_20, _rpl_22, _rpl_23(x2), _rpl_32, _rpl_33, _rpl_36
Ribosomal protein genes (small subunits)	_rps_2, _rps_3, _rps_4, _rps_7(x2), _rps_8, _rps_11, _rps_12(x2)#, _rps_14, _rps_15, _rps_16*, _rps_18, _rps_19
RNA Polymerase	_rpo_A, _rpo_B, _rpo_C1*, _rpo_C2
Ribosomal RNA genes	_rrn_4.5(X2), _rrn_5(X2), _rrn_16(X2), _rrn_23(X2)
Transfer RNA genes	_trn_I-CAU(x2), _trn_L-CAA(x2), _trn_V-GAC(x2), _trn_I-GAU(x2)*, _trn_A-UGC(x2)*, _trn_R-ACG(x2), _trn_N-GUU(x2), _trn_L-UAG, _trn_P-UGG, _trn_W-CCA, _trn_M-CAU, _trn_V-UAC*, _trn_F-GAA, _trn_L-UAA*, _trn_T-UGU, _trn_S-GGA, _trnf_M-CAU, _trn_G-GCC, _trn_S-UGA, _trn_T-GGU, _trn_E-UUC, _trn_Y-GUA, _trn_D-GUC, _trn_C-GCA, _trn_R-UCU, _trn_G-UCC*, _trn_S-GCU, _trn_Q-UUG, _trn_K-UUU*, _trn_H-GUG
ATP-dependent protease	_clp_P**
Maturase	_mat_K
Hypothetical chloroplast reading frames	_ycf_1(x2), _ycf2_(x2), _ycf_3**, _ycf_4
Acetyl-CoA carboxylase	_acc_D
C-type cytochrome synthesis gene	_ccs_A
Envelope membrane protein	_cem_A
Translational initiation factor	_inf_A

*genes with intron
#trans-spliced gene
