# Supplementary material for: Phylogeny and Taxonomic Synopsis of the Genus Bougainvillea (Nyctaginaceae)
Source: Plants (Basel). 2022 Jun 27;11(13):1700. doi: 10.3390/plants11131700 (PMC9269543; doi:10.3390/plants11131700)
Supplement: Supplementary file 1 [file plants-11-01700-s001.zip › Table S3.pdf]

Table S3. *Bougainvillea* samples included in the study

Sample name	Voucher	Place of Collection
Bougainvillea arborea Glaz.	Chen T. 2020031202, SZG	Shenzhen Fairy Lake Botanical Garden (originally from Miami)
Bougainvillea berberidifolia Heimerl.	Chen T. 20110122A, USZ	Saipina, Bolivia
Bougainvillea campanulata Heimerl.	Chen T. 20110122B, USZ	Saipina, Bolivia
Bougainvillea cultivar	Chen T. 2020031204 SZG	Shenzhen Fairy Lake Botanical Garden (originally from India)
Bougainville infesta Griseb.	Fortunato R. M. et al. 7286, MO	Santa Bárbara, Jujuy, Argentina
Bougainvillea luteoalba Heimerl ex E. Valenz.	Beck S.G. 5976, MO	Beni, Bolivia
Bougainvillea modesta Heimerl.	Chen T. 2011012101C, USZ	El Torno, Bolivia
Bougainvillea glabra Choisy	M.B.M. da Cruz 0001, NY	Ilhéus, Bahia, Brazil
Bougainvillea pachyphylla Heimerl ex Standl.	Sagastegui A. et al. 15924, MO	Chota, Cajamarca, Peru
Bougainvillea praecox Griseb.	Chen T. 2012063001, SZG	Jardim Botanico Plantarum, Nova Odes, Brazil
Bougainvillea peruviana Humb. & Bonpl.	Chen T. et al. 2014052606, SZG	on the way from Loja to Macara, Ecuador
Bougainvillea spinosa (Cav.) Heimerl.	1692, SI	Argentina
Bougainvillea stipitata Griseb.	Chen T. 2011012102, USZ	Samaipata, Bolivia
B. stipitata var. *grisebachiana* Heimerl.	Chen T. 2015061601, SZG	Kew, Richmond, UK (originally from Argentina)
