# Supplementary material for: Phylogeny and Taxonomic Synopsis of the Genus Bougainvillea (Nyctaginaceae)
Source: Plants (Basel). 2022 Jun 27;11(13):1700. doi: 10.3390/plants11131700 (PMC9269543; doi:10.3390/plants11131700)
Supplement: Supplementary file 1 [file plants-11-01700-s001.zip › Table S4.pdf]

Table S4. Data set included in the phylogenetic and comparative analyses of complete chloroplast DNA sequences (*Bougainvillea*, Nyctaginaceae)

	Family	Species	Accession Numbers
**Caryophyllales**	Petiveriaceae (outgroup)	*Seguieria aculeata*	NC_041418
		Rivina humilis	NC_041300
		Monococcus echinophorus	NC_041414
		Petiveria alliacea	NC_041417
**Caryophyllales**	Nyctaginaceae	*Salpianthus macrodontus*	MH286311
		Guapira discolor	MH286310
		Pisonia aculeata	MK397886
		Ascleisanthes obtusa	MH286321
		Nyctaginia capitate	MH286318
		Mirabilis jalapa	NC_041297
		Bougainvillea spectabilis	MK397858
		Bougainvillea spectabilis	MN315508
		Bougainvillea glabra	MN449976
		Bougainvillea stipitata var. *grisebachiana*	OM044392
		Bougainvillea arborea	OM044393
		Bougainvillea luteoalba	OM044394
		Bougainvillea berberidifolia	OM044395
		Bougainvillea stipitata	OM044396
		Bougainvillea spinosa	OM044397
		Bougainvillea modesta	OM044398
		Bougainvillea infesta	OM044399
		Bougainvillea campanulata	OM044400

	Bougainvillea glabra (Bahia)	MW123899
	Bougainvillea praecox	MW123900
	Bougainvillea peruviana	MW123901
	Bougainvillea pachyphylla	MW123902
	Bougainvillea cultivar	MW123903
