# Supplementary material for: Phylogeny and Taxonomic Synopsis of the Genus Bougainvillea (Nyctaginaceae)
Source: Plants (Basel). 2022 Jun 27;11(13):1700. doi: 10.3390/plants11131700 (PMC9269543; doi:10.3390/plants11131700)
Supplement: Supplementary file 1 [file plants-11-01700-s001.zip › Table S5.pdf]

Table S5. Morphological comparison of *Bougainvillea* species

Taxa	Habit	Branches	Thorn	Leaves	Bracts	Perianth	Anthocarp	Ovary	Stamens
Bougainvillea peruviana Humb. & Bonpl**.**	shrubs but sometimes scandent	sparsely puberulent or glabrate	simple, slender thorns, 1-2 cm long	alternate, glabrous, broadly ovate to suborbicular, 4-7 cm long	bright rose or peach, glabrous except for puberulent costa, 1.5-4 cm long, apex obtuse to rounded	glabrous, 15-18 mm long, perianth tube almost linear-oblong or slightly constricted, very slender	oblong-linear, glabrous, 10-20 mm long	oblong, ca. 4 mm long	6-7
Bougainvillea pachyphylla Heimerl ex Standl.	shrubs or small trees	finely puberulent when young	simple	alternate, thick, and somewhat leathery, finely, or densely puberulent, ovate, 3-6 cm long	Rose colored or light crimson, glabrate, broadly elliptic-ovate, apex obtuse	densely puberulent, 9-11 mm long	unknown		
Bougainvillea spinosa (Cav.) Heimerl	shrubs	sparsely puberulent when young	furcate or forked, 2-3 cm long	arranged in fascicles or brachyblasts, thick and fleshy, glabrous, narrowly spatulate, linear-spatulate or oblong-spatulate, 9-15 × 2-3 mm	yellowish white or pinkish, sparsely puberulent or glabrous, ovate-cordate to orbicular, about 12 mm long, membranous	glabrous or sparsely puberulent, reddish, 8-15 mm long, ovoid at the perianth base	anthocarp ovoid, 6-7 mm long	ovoid, ca. 4 mm long	5-8

Bougainvillea berberidifolia Heimerl	shrubs	glabrate but sparsely tomentulose when young	simple thorns, around 1-2 cm long	arranged in brachyblasts, lamina not fleshy, glabrate, elliptic-obovate, broadest in middle or slightly above, 1-2 cm long	pinkish but greenish white when dried, glabrous except for costa, 25-27 mm long, ovate, apex subobtuse or rounded, membranous	glabrous, reddish, perianth tube narrow, 11-14 mm long, ca. 1.5 mm wide, slightly constricted	unknown	oblong, 3.5-4 mm long	usually 5
Bougainvillea campanulata Heimerl	shrubs or trees	glabrous but slightly tomentulose when young	unarmed or armed with 2-3 cm simple thorns	alternate or in brachyblasts, glabrous but sometimes minutely hirtellous abaxially, elliptic-lanceolate to elliptic-oblong, broadest at or slightly below middle, 2-5 × 0.5-2 cm	yellowish green or white, glabrous or sparsely puberulent, ovate to elliptic-ovate, 8-20 mm long, 4-12 mm wide, apex obtuse	glabrous or hirtellous apically, greenish yellow, 6-7 mm long, perianth tube campanulate	obovoid	obovoid, ca, 2 mm	7-8
Bougainvillea infesta Griseb.	shrubs or small trees	densely pubescent but turns into glabrate upon maturity	unarmed or armed with simple thorns, 1-4 cm long	arranged in fascicles or brachyblasts, pubescent to densely puberulent or tomentulose abaxially, elliptic-lanceolate or elliptic-ovate, broadest at or below middle, 2-4 × 1-3 cm	greenish, densely pubescent, oblong-elliptic, 20-35 mm long, 11-16 mm wide, apex rounded or obtuse, membranous	densely pubescent or hirsute, greenish, 12-20 mm long, perianth tube fusiform at base	unknown	ovary navicular	5-6

Bougainvillea modesta Heimerl	Shrubs or trees	partially elongated, spreading obliquely, young parts tomentulose but glabrate upon maturity	unarmed	alternate, tomentose, elliptic to ovate-elliptic, 5-8 × 3-4 cm	pale yellow to whitish yellow, thin, moderately to densely puberulous on both surfaces, elliptic to elliptic-ovate, 15-17 mm long, acute at the apex, membranous	densely tomentulose, broad, yellowish brown perianth lobes, greenish-brown perianth tube, 2.5 mm wide, 10-11 mm long, almost straight or slightly constricted	ovoid to ellipsoid	oblong-ellipsoid	usually 6
Bougainvillea stipitata Griseb**.**	Shrubs or trees	young branches puberulent or tomentulose	unarmed or armed with simple thorns, 2-2.5 cm long	alternate, puberulent or glabrous, ovate, rhombic or ovate-lanceolate, 3.5-7 × 2-4 cm	greenish or pinkish purplish, sparsely or densely puberulent, broadly ovate or elliptic-ovate, 14-20 x 10-15 mm, apex acute or obtuse, papyraceous	puberulent, green or greenish yellow,11-25 mm, perianth tube very constricted in the middle, spindle-shaped at the base	spindle-shaped/fusiform, 12-15 mm long, minutely puberulent or glabrous	spindle-shaped/fusiform, 3 mm long	7-8
Bougainvillea praecox Griseb.	shrubs or trees	tomentulose but glabrate upon maturity	unarmed or sparsely armed with simple thorns, about 5 mm long	alternate, appressed tomentose abaxially but becoming glabrate, ovate to elliptic or ovate-elliptic, ca. 5-9 × 4-7 cm	white but greenish upon drying, densely puberulent, particularly on the costa and veins, broadly ovate, about 14 mm long, apex rounded, or obtuse	densely tomentulose, 9-11 mm long, oblong-ellipsoid or subfusiform at perianth base	ellipsoid or subfusiform	narrowly obconical, 3.5-4.5 × 1 mm	5-6

Bougainvillea spectabilis Willd.	scandent shrubs or woody vines	fulvous-villous, rarely glabrate	armed with numerous simple stout thorns, about 4 mm long	alternate, densely villous abaxially, ovate to suborbicular, 5-10 × 2-6 cm	purplish red, sparsely puberulent or short villous, ovate or oval, 2-4.5 cm long, abruptly acute at the apex	short villous, 15-30 mm, perianth lobes yellowish, perianth tube greenish, ellipsoid at the base	ellipsoid or oblong, 11-14 mm, short villous	ellipsoid or oblong	7-8
Bougainvillea glabra Choisy	scandent shrubs or woody vines	young branches pubescent but turns into glabrate upon maturity	armed with numerous simple, stout thorns	alternate, glabrate but very young leaves puberulent, ovate to ovate-lanceolate or elliptic, 4-10 cm long	purple to purplish-red, sparsely puberulent or glabrous, broadly ovate to oval, 1.5-4.5 cm long, acute or abruptly acuminate at the apex	sparsely to densely puberulent, 15-25 mm long, perianth lobes yellowish white, perianth tube greenish, oblong at the base	oblong, 8-15 mm long	oblong	7-8
Bougainvillea arborea Glaz.	Trees	young branches pubescent but glabrate upon maturity	unarmed or sparsely armed with simple thorns	alternate, puberulent but becoming glabrate as they mature, elliptic-lanceolate, 5-12 cm long	purple-pinkish, puberulent or densely puberulent, broadly ovate or elliptic, 2-4.5 cm long, apex acute or acuminate	sparsely to densely puberulent, 15-20 mm long, perianth lobes greenish yellow, perianth tube greenish, obconical to obturbinoid or fusiform at base	obconical, obturbinoid or fusiform, 8-10 mm long.	ovary fusiform or obturbinoid, ca. 4-5 mm long	7-8
